# SHARD: an improved method for staining and visualizing multiplex immunofluorescence in optically cleared postmortem human brain tissue

**DOI:** 10.3389/fnins.2024.1474617

**Published:** 2024-10-09

**Authors:** Grace A. Rosen, Daniel Kirsch, Raymond Nicks, Hunter Kelley, Rebecca Mathias, Kerry A. Cormier, Caroline A. Kubilus, Bryan Dec, Thor D. Stein, Victor E. Alvarez, Michael L. Alosco, Ann C. McKee, Bertrand R. Huber

**Affiliations:** ^1^VA Boston Healthcare System, US Department of Veterans Affairs, Boston, MA, United States; ^2^Department of Neurology, Boston University Chobanian and Avedisian School of Medicine, Boston, MA, United States; ^3^National Center for PTSD, US Department of Veterans Affairs, Boston, MA, United States; ^4^Department of Pathology and Laboratory Medicine, Boston University Chobanian and Avedisian School of Medicine, Boston, MA, United States; ^5^Boston University Alzheimer's Disease Research Center and Boston University CTE Center, Boston, MA, United States; ^6^VA Bedford Healthcare System, US Department of Veterans Affairs, Bedford, MA, United States; ^7^Department of Neurology, Boston Medical Center, Boston, MA, United States; ^8^Department of Anatomy and Neurobiology, Boston University Chobanian and Avedisian School of Medicine, Boston, MA, United States

**Keywords:** optical clearing, antigen retrieval, volumetric imaging, 3D confocal imaging, 3D confocal laser scanning microscopy, human brain banking, immunofluorescence

## Abstract

Postmortem human brain tissue is a critical resource for studying neurodegenerative disease, providing critical insights into cellular morphology, pathology, and network connectivity. To improve standard microscopy and enable high-resolution, three-dimensional (3D) images of tissues at the subcellular level, tissue-clearing methods have been developed. These 3D images allow for the analysis of large regions of interest and can be used to study structural and spatial changes that occur during neurodegeneration. Additionally, 3D imaging facilitates the visualization of whole-cell morphology, especially in cells with long processes that would otherwise be truncated in single-plane images. Human brain tissue is especially challenging for tissue clearing due to the abundance of lipids in myelin and the need for optimal fixation and low postmortem intervals. Formaldehyde-based fixatives, commonly used in preserving tissue, hinder antibody binding by crosslinking important antibody epitopes, and fluorescent microscopy requires the incorporation of fluorescent labels through passive diffusion or electrophoresis. Recent studies have focused on optimally fixed human brain tissue with short postmortem intervals, limiting the general applicability of these methods. To address these challenges, we developed SHARD (SHIELD, antigen retrieval, and delipidation), a simple and widely applicable method for clearing and labeling human brain tissue, which can be applied to long-term banked human brain tissue preserved in formaldehyde. SHARD is a novel addition to the SHIELD tissue clarification method, combining antigen retrieval, tissue clearing, and staining of 200-μm sections from long-term banked human brain tissue. The SHARD method is effective for postmortem intervals (PMIs) ranging from 10 to 72 h in multiple neurodegenerative diseases and control samples. In this study, we demonstrate that the SHARD method significantly enhances the immunostaining of glial fibrillary acidic protein (GFAP), an astrocytic cytoskeletal marker. Overall, the combination of antigen retrieval and tissue delipidation holds great potential for achieving detailed 3D immunostaining in long-term formaldehyde-fixed postmortem human brain tissue, opening new avenues for research and discovery.

## 1 Introduction

Postmortem human brain tissue is an invaluable resource for studying microscopic structural changes associated with neurodegenerative and psychiatric disorders. Three-dimensional tissue imaging offers advantages over standard microscopy by enabling improved visualization of cellular morphology and spatial relationships between cells. However, this technique requires optically transparent tissue and the accessibility of target epitopes to antibodies, both of which present technical challenges.

The three major approaches for volumetric imaging are hydrophobic, hydrophilic, and hydrogel-based clearing methods. Hydrophobic methods such as 3DISCO lead to well-preserved cleared organs within a few days, with downsides of tissue shrinkage, reagents that include toxic organic solvents, and the possibility of fluorescent signal degradation over time due to protein instability in clearing solutions (Ertürk et al., 2012; Ueda, 2020). Hydrophilic clearing methods such as CUBIC have the advantage of preserving protein structure and function due to their use of water-soluble reagents (Matsumoto et al., 2019). Hydrogel-based methods offer additional structural stabilization through crosslinking proteins, nucleic acids, and hydrogel monomers that are in place in preparation for lipid removal (Park et al., 2019). Compared to formalin fixation alone, hydrogel-embedding of brain tissue also results in minimal protein loss during clearing (Chung et al., 2013).

Due to the advantages of hydrogel-based methods, we opted to use the SHIELD protocol (stabilization under harsh conditions via intramolecular epoxide linkages to prevent degradation). After the SHIELD procedure, lipids are washed from the tissue using sodium dodecyl sulfate (SDS). If actively clearing, SDS is driven into the tissue with an electric current (Kim et al., 2015).

The fully cleared tissue can be fluorescently labeled and incubated in an index-matching solution so that its refractive index matches that of a glass cover slip. Previous SHIELD-based studies have successfully characterized the morphology and spatial distribution of cells in tissue sections up to 8 mm thick (Chung and Deisseroth, 2013; Ueda, 2020); tissue thickness is limited by the index of refraction inhomogeneity, which blurs images, and by the optical limitations of the microscope. Another challenge to 3D imaging is laser absorption and scattering by pigmented tissue features such as melanocytes.

Tissue clearing and fluorescent labeling have been successfully employed to image whole mouse brains, approximately 13.2 mm × 11.4 mm × 8 mm (Chung and Deisseroth, 2013; Kim et al., 2015; Yun et al., 2019; Wang et al., 2020) and many human organs, including kidney (approximately 12 × 8 × 5 cm), pancreas (approximately 11.5 × 4 × 2 cm), heart (approximately 12 × 8 × 6 cm), lung (approximately 13 × 8 × 0.5 cm), spleen (approximately 13 × 8 × 4 cm), and 2 mm thick brain 6 cm × 8 cm coronals (Ku et al., 2020; Mai et al., 2022; Shi et al., 2022). Previous studies clearing postmortem human brain sections have required minimal fixation several months of clearing (Chung et al., 2013; Liebmann et al., 2016; Liu et al., 2016; Phillips et al., 2016; Lai et al., 2018; Morawski et al., 2018), or the use of 100 μm sections, cleared over a few weeks, that resulted in warping of the tissue surface (Murray et al., 2015). The utility of tissue clearing has been demonstrated through the qualitative visualization of Alzheimer’s disease (AD) amyloid plaques in humans (Ando et al., 2014). Moreover, 2–5 mm human brain sections have been successfully immunostained and volumetrically visualized with a hydrophobic clearing method (Hildebrand et al., 2019).

One barrier to successful immunofluorescent staining in postmortem human brain tissue is signal specificity. Postmortem human tissue is autofluorescent due to sources such as lipids, lipofuscin, hemoglobin, hemosiderin, collagen, and myelin. Recent protocols have added a photobleaching step to reduce background signal from autofluorescence (Duong and Han, 2013; Ku et al., 2020). Furthermore, antigen sites for stains may be masked by formaldehyde crosslinking. Formaldehyde fixation results in inter- and intramolecular crosslinking that results in redistribution of electrostatic charges and physical hindrance of the antigen site availability (Rait et al., 2004; Boenisch, 2006). Antigen retrieval is a process in which the chemical crosslinking that happens between formaldehyde and amino acids is reversed, making epitopes available for immunochemistry (D’Amico et al., 2009). Protocols for antigen retrieval involve incubating tissue in acidic or basic solutions at near boiling heat, resulting in increased staining quality. The utility of antigen retrieval has been demonstrated in pre-fixed frozen mouse tissue (Ino, 2003), frozen human tissue (Alelú-Paz et al., 2008), as well as thin formaldehyde-fixed paraffin-embedded (FFPE) 3 μm to tens of μm sections of human tissue (Namimatsu et al., 2005; Shi et al., 2007). There is a growing body of literature on optimizing antigen retrieval in thicker sections than light scattering in FFPE allows (Evers and Uylings, 1994; Jiao et al., 1999). Alkaline antigen retrieval has been successfully implemented in 500 μm sections of postmortem human brain tissue to visualize neuronal, glial, and vasculature markers (Pesce et al., 2022). Sodium dodecyl sulfate (SDS) has also been used as a medium for antigen retrieval for tissue-clearing organoids and human organs (Messal et al., 2021).

In this study, we introduced SHARD (SHIELD, antigen retrieval, and delipidation), a method by which SHIELD-treated tissue undergoes citrate-based antigen retrieval, delipidation with SDS, and stained for three-dimensional rendering of targets of interest via fluorescent antibodies and small-molecule dyes. SHARD works well across a range of postmortem intervals and disease states and can also be used in a multiplex staining with up to seven colors. We tested it alongside a SHIELD-only protocol and with an additional photobleaching step. To the best of our knowledge, this is the first instance of quantitative analysis of different tissue-clearing treatments across various neurodegenerative disease states using long-term formaldehyde-fixed human tissue3W. The protocol we developed provides a method for tissue clearing and antigen unmasking that is compatible with long-term formaldehyde-fixed human brain tissue and works with the majority of autopsy tissue currently available from brain banks. SHARD offers key benefits of scalability, simplicity, and the ability to stain and image up to six targets, and it can be widely applied to render markers of interest in three dimensions in a few weeks’ time.

## 2 Materials and equipment

### 2.1 Reagents

- Paraformaldehyde (PFA, Sigma-Aldrich # S812315 325).
- Sodium Phosphate, dibasic (Sigma-Aldrich # S9763).
- Sodium phosphate, monobasic (Sigma-Aldrich # S8282).
- Sodium-m-periodate (Sigma-Aldrich # 311448).
- L Lysine (Sigma-Aldrich # L8662).
- Sodium Hydroxide (Fisher Scientific # S318).
- Potassium phosphate, dibasic (Sigma-Aldrich # P3786).
- SHIELD Kit (LifeCanvas # SH-250, includes SHIELD-Buffer Solution, SHIELD-Epoxy Solution, and SHIELD ON).
- Triton X-100 (Fisher Scientific # BP151).
- Normal Donkey Serum (Jackson ImmunoResearch # NC9624464).
- Sodium chloride (Sigma-Aldrich # S5886).
- Agarose, low gelling (Sigma-Aldrich # A9414).
- Citric acid (Sigma-Aldrich # C7129).
- Sodium citrate tribasic dihydrate (Sigma-Aldrich # S4641).
- Sodium dodecyl sulfate (Fisher Scientific # BP166).
- Boric acid (Sigma-Aldrich # B7901).
- Sodium sulfite anhydrous (Fisher Scientific # S430).
- Tris Base (Fisher Scientific # BP154).
- EDTA 0.5 M, pH 8 (Invitrogen # 15575–038).
- Tween (Thermo Scientific # J20605).
- Hydrogen peroxide (Fisher Scientific # H325).
- Sucrose (Sigma-Aldrich # S0389).
- 10% buffered formalin phosphate (Fisher Scientific # SF100).
- Sodium azide (Sigma-Aldrich # S2002).
- Tomato lectin DyLight 488 conjugate (Vector Laboratories # DL-1174).
- Primary antibodies: see Table 1.
- Alexa Fluor^®^ 750-Anti-Mouse (abcam # ab175738).
- Alexa Fluor^®^ 647-Anti-Mouse (Jackson ImmunoResearch # 715–605-150).
- Alexa Fluor^®^ 647-Anti-Rabbit (Jackson ImmunoResearch # 711–605-152).
- Alexa Fluor^®^ 594-Anti-Chicken (Jackson ImmunoResearch # 703–585-155).
- Alexa Fluor^®^ 594-Anti-Goat (Jackson ImmunoResearch # 705–585-003).
- Cy3-Anti-Goat (Jackson ImmunoResearch # 705–165-147).
- Cy3-Anti-Rabbit (Jackson ImmunoResearch # 711–167-003).
- Cy3-Anti-Chicken (Jackson ImmunoResearch # 703–166-155).
- Alexa Fluor^®^ 488-Anti-Mouse (Jackson ImmunoResearch # 715–545-150).
- Alexa Fluor^®^ 488-Anti-Rabbit (Jackson ImmunoResearch # 711–545-152).
- EasyIndex (LifeCanvas # EI-500-1.52).

**Table 1:** Detailed information on the primary antibodies used.

Figure 1D	Target	Primary antibody	Host	Amount in 1 mL	Vendor ID	Specificity (vendor)
Glial markers						
Figures 1B,F,J Figures 2B,F,J Figures 6B,F,J,N	Astrocytes	Aquaporin 4, C-terminus	Rabbit	12 μg	Millipore AB3594	Immunogen based on rats, reacts with humans, mice, and rats
Figures 1C,G,K Figures 2C,G,K Figures 6C,G,K,O Figure 7A2 Figure 8E Figure 9E	Astrocytes	GFAP (cocktail)	Mouse	10 μg	BioLegend SMI 26	Reacts with humans, mice, rats
Figure 7A1	Astrocytes	GFAP	Camel nanobody	0.75 μg	Synaptic Systems N3802	Recombinant based on humans. Reacts with humans, rats, mice
Figure 7A2	Astrocytes	Glutamine Synthetase	Rabbit	6.8 μg	Abcam ab49873	Immunogen based on a mouse reacts with humans and rats
Figure 7A3	Microglia and macrophage	Iba1	Rabbit	0.75 μg	Wako 019–19,741	Humans, mice, rats
Figure 7A4 Figure 8A Figure 9A	microglia and macrophage	Iba1	goat	1 μg	Wako 011–27,991	Mice, rat
Neuronal markers						
Figure 7B1 Figure 8B Figure 9B	Neurons	Map2	Chicken	1.25 μg	abcam ab5392	Recombinant based on humans. Reacts with humans, mice, rats, sheep, cows, dogs, monkeys, marmosets, alpaca
Figure 7B2	Neurons	Map2	Rabbit	2 μg	Sigma-Aldrich HPA 12828	Reacts with humans
Figure 7B3	Neurons	Neurofilament (pan-neuronal, cocktail)	Mouse	1 μg	BioLegend 837,802	Humans, mice, rats
Figure 7B4	Neurons	Map2	Camel nanobody	0.5 μg	Millipore MAB3418	Immunogen based on a mouse reacts with humans, chickens, mice, rats, Xenopus
Figure 7B4	Intercellular junction	Connexin-43	Rabbit	0.25 μg	abcam ab11370	Recombinant based on humans. Reacts with humans, mice, rats, hamsters, cows, dogs, pigs, monkeys
Figure 7B5 Figure 8C Figure 9C Figure 7B5	Myelin sheath	MBP	Rabbit	0.5 μg	GeneTex	Humans, mice, rats, fish
Figure 7B5	Myelin sheath	Casper	Mouse	3 μg	Abcam ab252535	Humans, mice, rats
Figure 7B6	Catechol-aminergic neuron	Tyrosine hydroxylase	Rabbit	1.5 μg	Abcam ab112	Recombinant based on rats, reacts with rats and humans
Disease-related markers						
Figure 7C1 Figure 8D Figure 9D	Alzheimer’s Disease (AD) plaque	β-amyloid	n/a	250 μM	Tocris 4,920	Mice and humans
Figure 7C2	Hyper-phosphorylated tau	pSer202, pThr205 (AT8)	Mouse	2.5 μg	Thermo Fisher Scientific MN 1020	Immunogen based on humans, reacts with humans and mice
Figure 7C3	Hyper-phosphorylated tau	pThr231	Rabbit	2.5 μg	AnaSpec AS-55313	Immunogen based on humans reacts with humans

### 2.2 Materials/equipment

- Vibratome (Precisionary Instruments, Inc. # VF-700-0Z).
- Microscope Slides, Precleaned (Fisher Scientific # 12–550-15).
- No. 1.5 Coverglass, 22 × 30 mm (Corning # 2980–223).
- Zeiss 880 LSM Confocal Microscope with Airyscan and 20x PLAN APO Lens, 0.8 numerical aperture (NA), (Zeiss).
- Nikon Ti2 Eclipse Crest V2 spinning disc confocal microscope with 60x PLAN APO λD, 1.42 NA oil, and 100x CFI PLAN APO D 1.45 NA oil objective lenses, as well as a 20x CFI PLAN APO D, 0.8 NA lens.
- Nikon AX-R confocal microscope with 40x CFI PLAN APO silicone oil lens, 1.25 NA with cover glass thickness correction collar set to 0.17 mm, 60x CFI PLAN APO λD, 1.42 NA oil lens, 20x CFI PLAN APO λD, 0.8 NA lens, or 4x CFI PLAN APO λD 0.20 NA lens, equipped with one multi-alkaloid (MA) detector, two tunable GaAsP detectors, one non-tunable GaAsP detector, and seven laser lines: 405, 488, 514, 561, 594, 633, and 730 nm.

### 2.3 Solution formulations

- Periodate-Lysine-Paraformaldehyde (PLP): Heat 1 L of deionized water to 60°C. Add 80 g of paraformaldehyde and 44 g of sodium phosphate dibasic. Cool the solution to room temperature using a water bath. Add 12 g of sodium phosphate monobasic and 8.6 g L of L-lysine Filter the solution and add deionized (DI) water to reach a total volume of 4 L. Adjust the pH to 7.4 using sodium hydroxide.
- SHIELD OFF solution (per 25 mm × 25 mm × 3 mm sample): Mix 5 mL of DI water, 5 mL of SHIELD-buffer solution, and 10 mL of SHIELD-epoxy solution.
- 25x phosphate-buffered saline (PBS): Dissolve 188 g of potassium phosphate dibasic in approximately 800 mL of deionized water, heating as needed. Add sodium phosphate monobasic and sodium chloride and stir until fully dissolved. Cool the solution and dilute it to a final volume of 1 L.
- PBS: Mix 40 mL of 25x stock solution with 960 mL of deionized water.
- 10 mM citrate: Mix 14 mL of 0.1 M citric acid with 86 mL of 0.1 M sodium citrate. Add 900 mL of deionized water to achieve a total volume of 1 L.
- 30% sucrose: Dissolve 300 g of sucrose into 1 L of PBS.
- Passive clearing buffer: Prepare a buffer containing 300 mM sodium dodecyl sulfate (SDS), 10 mM boric acid, and 100 mM sodium sulfite. Adjust the pH to 9.
- PBST: Add 4 mL of Triton X-100 to 996 mL of PBS to make 1 L of PBST.
- Alkaline antigen retrieval solution: Prepare a solution with 10 mM Tris base, 1 mM EDTA, and 0.05% Tween. Adjust the pH to 9.
- 5% donkey serum (DKS): Dilute 50 μL of donkey serum in 950 μL of PBST.
- Primary antibody solution: Add the specified amounts of primary antibodies from Table 1, along with 10 μL of DKS, and adjust the volume to 1 mL with PBST.
- Secondary antibody solution: Prepare the secondary antibody of choice at a 2:1 molar ratio of secondary to the primary antibody, add 10 μL of DKS, and adjust the volume to 1 mL with PBST.
- 4% PFA 1 Prepare a 1:2.5 dilution of 10% PFA stock in PBS to make 4% PFA.
- 0.02% sodium azide tissue storage solution: Dissolve 0.2 g of sodium azide in 1 L of PBS.

### 2.4 Software and computer

- Zen (Black Edition) with Airyscan Deconvolution (Zeiss).
- Nikon Elements Advanced Research 6.02.01 (Nikon).
- Imaris 10.0 (Oxford Instruments) running on a Windows 10 system with an Intel^®^ Xeon^®^ Gold 5,218 CPU @ 2.30 GHz and 2.29 GHz processors, 192 GB of RAM, and an NVIDIA RTX A6000 Graphics Card.
- Arivis Pro, running on a Windows 11 system with an AMD Ryzen Threadripper PRO 5995WX with 64 cores @ 2.70 GHz, 512 GB of RAM, and an NVIDIA RTX A6000 Graphics Card.

## 3 Methods

### 3.1 Study design and brain donors

Autopsy participants included 21 brain donors from three brain banks housed at VA Boston Healthcare System with harmonized neuropathological processing protocols and diagnostic procedures: Understanding Neurological Injury and Traumatic Encephalopathy (UNITE, *n* = 13), Boston University Alzheimer’s Disease Research Center (ADRC *n* = 3), and the National Posttraumatic Stress Disorder Brain Bank (NPBB, *n* = 5). UNITE’s objective is to characterize the neuropathology and clinical-pathological correlates of CTE and repetitive head impacts (Mez et al., 2015; Alosco et al., 2021). The ADRC longitudinally follows its participants to collaborate with the National Alzheimer’s Coordinating Center to study Alzheimer’s Disease (AD) and related dementia (Gavett et al., 2012). The NPBB facilitates research on the cause, progression, and treatment of PTSD (Friedman et al., 2017). Consents for brain donation and research participation were provided by the donors’ next of kin. Institutional review boards from the Boston University Medical Center and VA Boston Healthcare System approved brain donation, postmortem clinical record review, neuropathological evaluation, and clinical interviews with donor family members. Cases were coded as “u,” “p,” or “a,” depending on whether they came from the UNITE, NPBB, or ADRC brain banks (Table 2).

**Table 2:** Demographics and neuropathological diagnoses of the cases studied.

Case	Age (yrs.)	Sex (M/F)	AD diagnosis	CTE diagnosis	PMI (h)	Fixation year	Brain bank	Cause of death
u1	35	M	No	Yes	10	2016	UNITE	Injury
u2	72	M	Yes	No	19.3	2016	UNITE	AD
u3	65	M	Yes	No	72	2017	UNITE	Dementia
u4	76	M	Yes	No	42	2017	UNITE	Dementia
u5	57	M	No	Yes	16	2017	UNITE	Suicide
u6	84	M	No	Yes	50	2017	UNITE	Cardiovascular disease
u7	81	M	Yes	No	25	2018	UNITE	AD
p1	71	M	No	No	31.5	2018	NPBB	Mucus plug
p2	64	M	No	No	27.13	2019	NPBB	Cardiorespiratory
p3	61	M	No	No	22.45	2020	NPBB	Liver disease
p4	61	M	No	No	26.78	2020	NPBB	Myocardial infarction
u8	85	M	Yes	No	24.3	2019	UNITE	AD
u9	64	M	No	Yes	24	2011	UNITE	Dementia
u10	65	M	No	No	15.08	2019	UNITE	Lethal injection
u11	66	M	No	No	44	2021	UNITE	Pneumonia
p5	67	M	No	No	24.22	2019	NPBB	Congestive heart failure
a1	86	M	No	No	15	2016	ADRC	Congestive heart failure
a2	93	F	Yes	No	14	2014	UNITE	Unknown
a3	86	M	Yes	No	6.25	2016	ADRC	Unknown
u12	46	M	No	Yes	54	2019	UNITE	Cirrhosis
u13	46	M	No	No	Unknown	2016	UNITE	Dementia

We examined 11 cases for quantification of the effectiveness of the three different treatment conditions and 10 further cases for staining with additional antibodies. Cases u1-7 and p1-4 were used for the quantification of tomato lectin, aquaporin-4, and GFAP staining. The postmortem interval (PMI) of these cases ranged from 10 to 72 h. The PMI here is defined by the difference between the time of death and brain removal. The cases had been stored at 4° C in Periodate-Lysine-Paraformaldehyde (PLP) for up to five years at the time of staining. There was a mix of healthy controls (*n* = 4), CTE cases (*n* = 3), and AD cases (*n* = 4). The 10 cases stained with additional antibodies had PMI values ranging from 6.25 to 54 h and included cases that had been stored in PLP for 1–10 years at the time of staining.

### 3.2 Clinical assessment and diagnosis

Demographic information, medical history, and other antemortem clinical variables were obtained during retrospective clinical evaluation with informants for all brain donors and included a detailed assessment of repetitive head impact exposure and traumatic brain injury history for the UNITE brain bank (Mez et al., 2015; Alosco et al., 2021). Tissue acquisition, processing, diagnostic assessment protocols, ethical considerations, governance, and oversight of the VA National PTSD Brain Bank were previously described (Friedman et al., 2017). Details on the procedures of the BU ADRC brain bank are available in prior publications (Gavett et al., 2012; Alosco et al., 2021). Diagnoses for Alzheimer’s disease were assigned according to the National Institute on Aging-Alzheimer’s Association guidelines (Montine et al., 2012).

### 3.3 Tissue processing and pathological assessment

Postmortem brain tissue was fixed in periodate-lysine-paraformaldehyde (PLP) for at least 3 months at 4°C. Neuropathological assessment was performed using procedures previously established (Vonsattel et al., 2008; Mez et al., 2015). Neuropathological evaluations were made by board-certified neuropathologists (ACM, TDS, BRH) according to published diagnostic criteria and were kept blinded to antemortem clinical information (Mez et al., 2015).

### 3.4 SHIELD post-fixation

PLP-fixed dorsolateral prefrontal cortex (DLF, Brodmann area 46) tissue blocks were harvested with a 16 × 16 mm^2^ leather punch with a thickness of 3 mm. Tissue harvesting was based on a standardized blocking scheme that is used in all cases. The 16 × 16 × 3 mm tissue sections were incubated in SHIELD OFF solution (LifeCanvas SHIELD-buffer solution, LifeCanvas SHIELD-Epoxy solution, and deionized water) for 3 days with shaking at 4°C (LifeCanvas, Cambridge, MA). Sections were transferred to LifeCanvas SHIELD ON buffer and incubated for 24 h. with shaking at 37°C and stored in phosphate-buffered saline (PBS) with 0.02% sodium azide for up to several weeks. SHIELD-preserved sections were embedded in 2% agarose gel and cut into 200 μm slices using a vibratome (VF-700-0Z Microtome, Precisionary Instruments, Inc., Natick, MA). Slices were stored in PBS with 0.02% sodium azide for up to a few months.

### 3.5 Tissue clearing and antigen retrieval

A summary of the three tissue treatment conditions quantified can be found in Table 3.

**Table 3:** Summary of tissue treatment conditions.

	SHIELD only	SHARD	SHARD+PB
SHIELD preservation	Yes	Yes	Yes
Citrate antigen retrieval	No	Yes	Yes
Delipidation	Yes	Yes	Yes
Photobleaching	No	No	Yes

#### 3.5.1 SHIELD only condition

For tissue subjected to the “SHIELD only” treatment, the delipidation step was started immediately after the SHIELD treatment. Delipidation consists of overnight incubation at 37°C with light shaking in a passive clearing buffer. Slices were subsequently washed twice with PBST at room temperature (RT) with light shaking to wash out the SDS.

#### 3.5.2 SHARD condition

Tissue slices that underwent antigen retrieval were incubated with 10 mM citrate buffer overnight at 4°C. The next day, slices were incubated in 10 mM citrate buffer at 95°C for 15 min before transferring to 30% sucrose in PBS at 4°C, where they remained for a few hours or overnight until they sank in the solution. Slices were then delipidated overnight as described above. The “SHARD” condition refers to tissue slices that were stained immediately after this point.

#### 3.5.3 SHARD+PB condition

After antigen retrieval with citrate and delipidation, sections were photobleached with white light LEDs. Sections that were photobleached were done so for 5 days in PBST, as previously described (Ku et al., 2020). This treatment is referred to as “SHARD+PB” throughout the paper.

**Figure 1:**
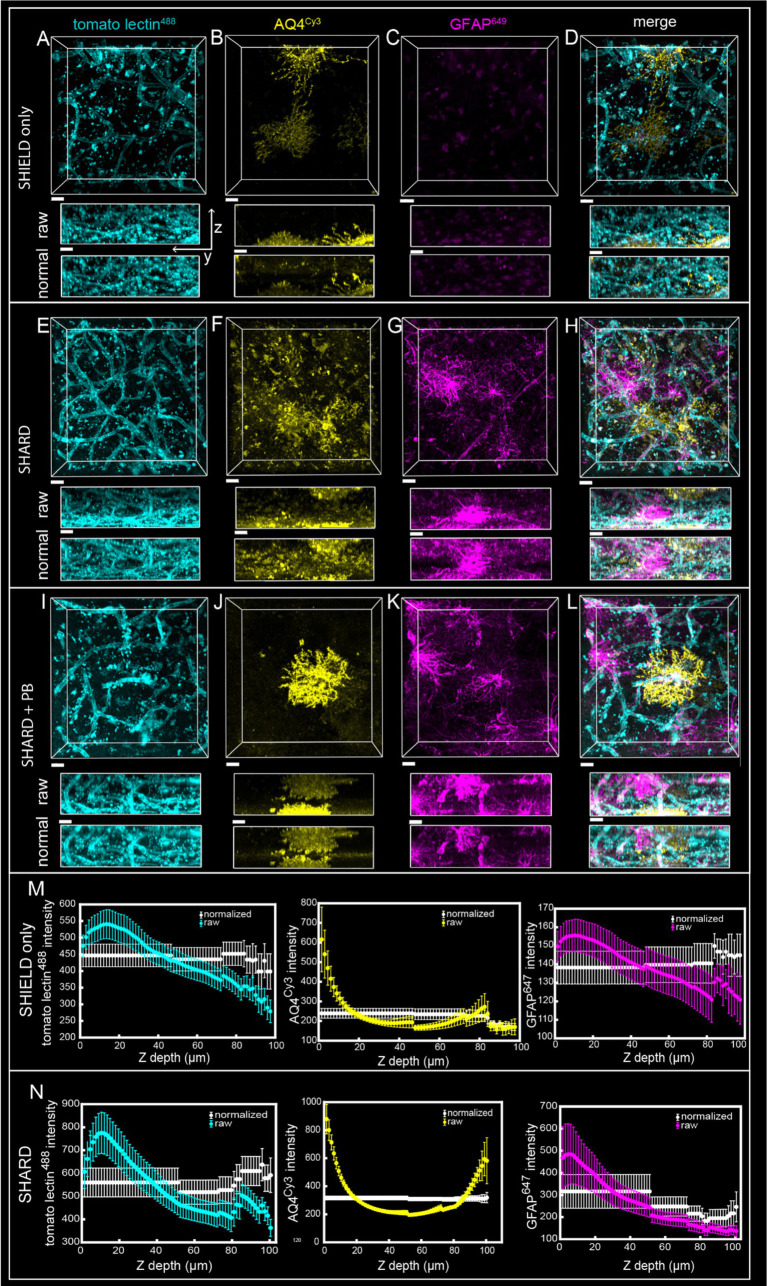
Comparison of SHIELD only, SHARD, and SHARD+PB conditions in the gray matter. 3D image stacks of immunofluorescent stains in the DLF gray matter resulting from the three experimental conditions: **(A–D)** SHIELD only, **(E–H)** SHARD, **(I–L)** SHARD+PB. The three stains shown are tomato lectin^488^ **(A,E,I)**, anti-AQ4^Cy3^ **(B,F,J)**, and anti-GFAP^647^ **(C,G,K)**. The overlay of all three is shown in **(D,H,L)**. All images are from the same 64-year-old case with no neurodegenerative disease. Scale bar = 20 μm, 72 μm Z-stacks. Display parameters for the dynamic range were held constant for each stain between conditions. **(M,N)** Intensity vs. imaging depth plots averaged over the 11 quantified cases for tomato lectin^488^ (left), AQ4^Cy3^ (middle), and GFAP^647^ (right). Raw pixel intensity (colored) and normalized pixel intensities (white) are both plotted for SHIELD-only tissue treatment **(M)** and SHARD tissue treatment **(N)**.

**Figure 2:**
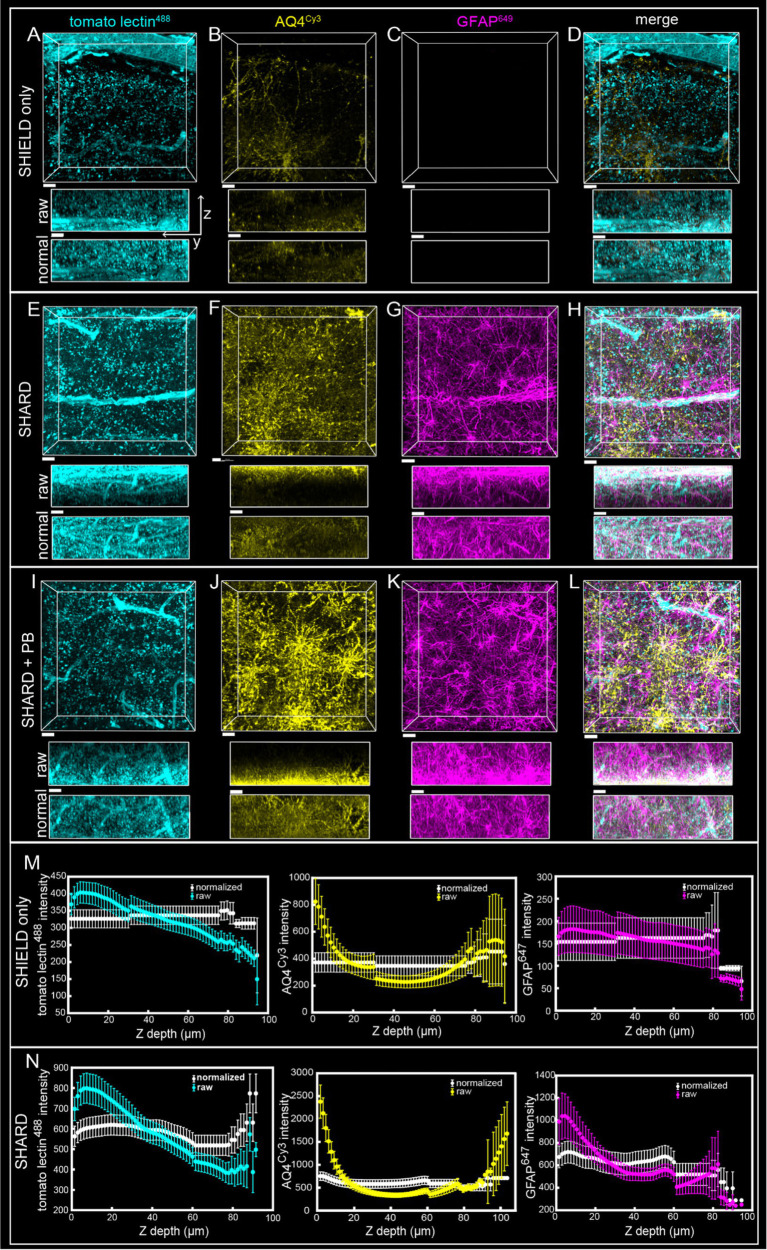
Comparison of SHIELD only, SHARD, and SHARD+PB conditions in the white matter. 3D image stacks of immunofluorescent stains in the DLF white matter resulting from the three experimental conditions: **(A–D)** SHIELD only, **(E–H)** SHARD, and **(I–L)** SHARD+PB. For each image, the views displayed are xy (top) and yz (middle and bottom). The middle yz view shows raw pixel intensity, whereas the bottom yz view shows pixel intensity after running normalized layers preprocessing in Imaris. The three stains shown are tomato lectin^488^ **(A,E,I)**, anti-AQ4^Cy3^ **(B,F,J)**, and anti-GFAP^647^ **(C,G,K)**. The overlay of all three is shown in **(D,H,L)**. Images are taken using tissue donated by the same individual as Figure 1. Scale bar = 20 μm, 72 μm Z-stacks. Display parameters for the dynamic range were held constant for each stain between conditions. **(M,N)** Intensity vs. imaging depth plots averaged over the 11 quantified cases for tomato lectin^488^ (left), AQ4^Cy3^ (middle), and GFAP^647^ (right). Raw pixel intensity (colored) and normalized pixel intensities (white) are both plotted for SHIELD-only tissue treatment **(M)** and SHARD tissue treatment **(N)**.

**Figure 3:**
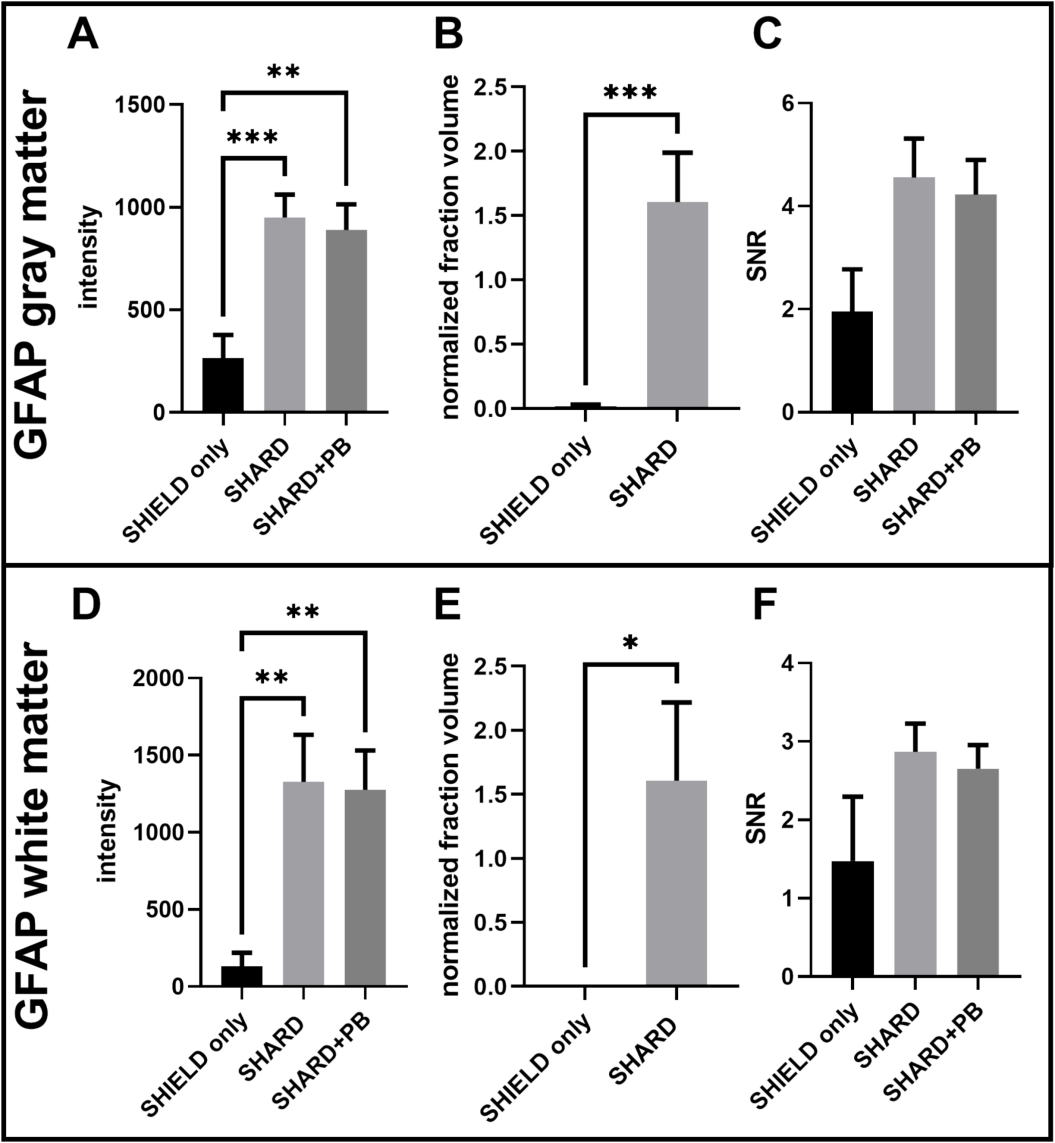
Quantification of GFAP^647^ staining across the three experimental conditions in DLF tissue. **(A)** GFAP^647^ signal intensity in the gray matter, **(B)** GFAP^647^ normalized detected fraction volume in the gray matter, **(C)** GFAP^647^ signal-to-noise ratio in the gray matter, **(D)** GFAP^647^ signal intensity in the white matter, **(E)** GFAP^647^ normalized detected fraction volume in the white matter, and **(F)** GFAP^647^ signal-to-noise ratio in the white matter. *p < 0.05, **p < 0.01, ***p < 0.001.

**Figure 4:**
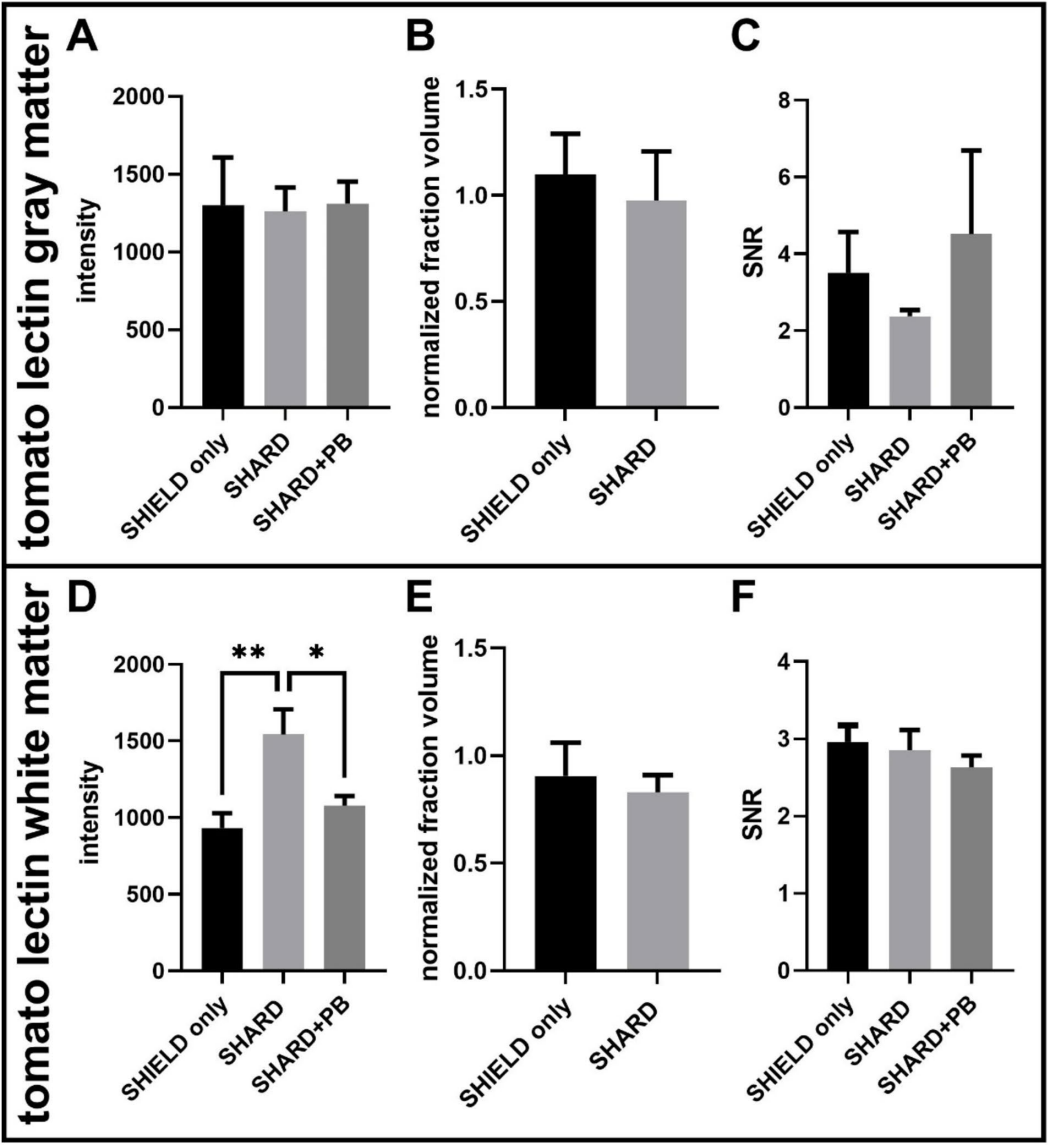
Quantification of tomato lectin^488^ staining across the three experimental conditions in DLF tissue. **(A)** Tomato lectin^488^ signal intensity in the gray matter, **(B)** tomato lectin^488^ normalized detected fraction volume in the gray matter, **(C)** tomato lectin^488^ signal to noise ratio in the gray matter, **(D)** tomato lectin^488^ signal intensity in the white matter, **(E)** tomato lectin^488^ normalized detected fraction volume in the white matter, and **(F)** tomato lectin^488^ signal to noise ratio in the white matter. *p < 0.05, **p < 0.01. For each condition, the number of cases was 11 cases.

**Figure 5:**
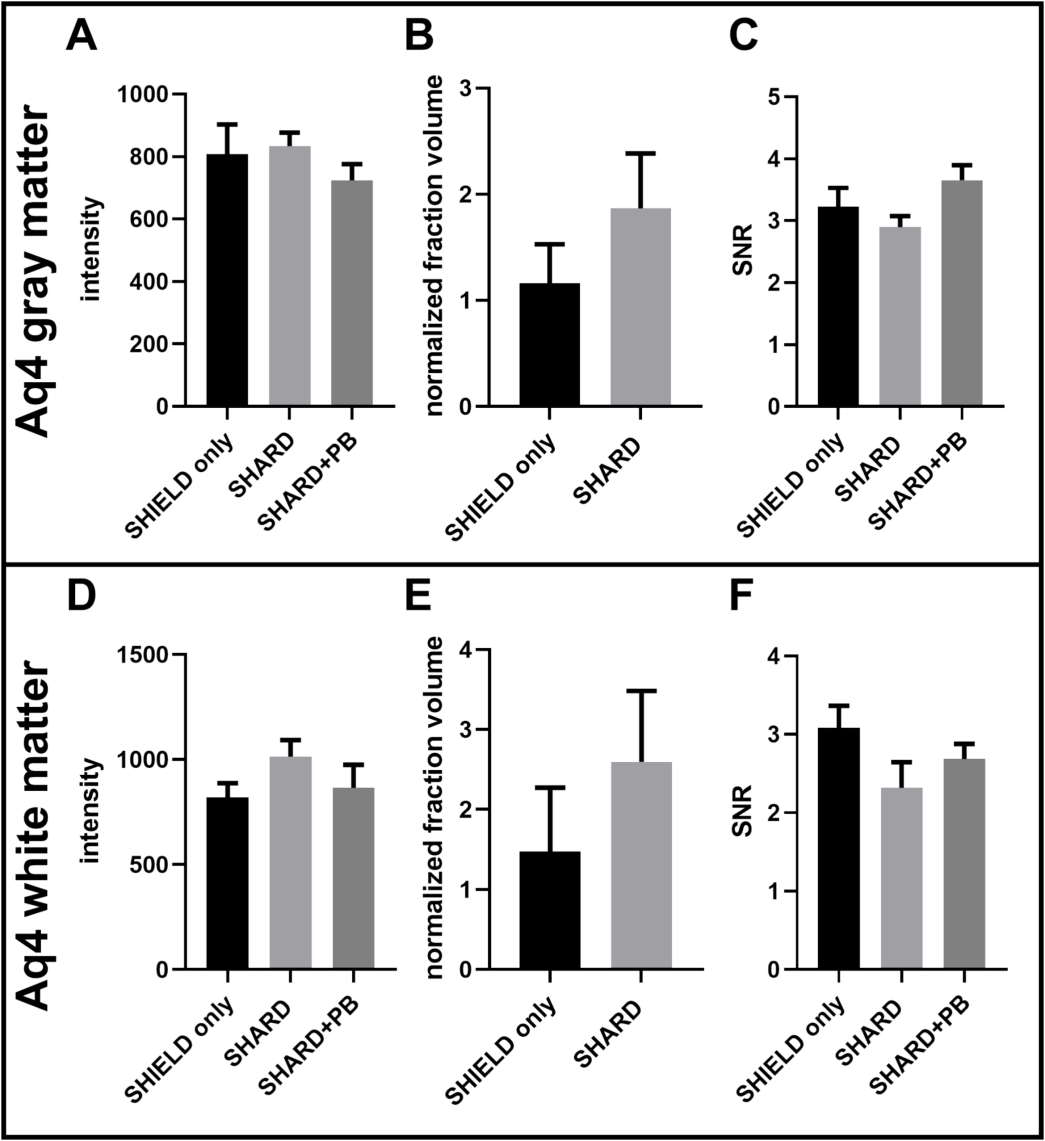
Quantification of Aq4^Cy3^ staining across the three experimental conditions in DLF tissue. **(A)** AQ4 ^Cy3^ signal intensity in the gray matter, **(B)** AQ4 ^Cy3^ normalized detected fraction volume in the gray matter, **(C)** AQ4 ^Cy3^ signal to noise ratio in the gray matter, **(D)** AQ4 ^Cy3^ signal intensity in the white matter, **(E)** AQ4 ^Cy3^ normalized detected fraction volume in the white matter, and **(F)** AQ4^Cy3^ signal to noise ratio in the white matter. For each condition, *n* = 11 cases.

In summary, these three conditions will be called “SHIELD only” (delipidation without antigen retrieval or photobleaching), “SHARD” (SHIELD and antigen retrieval), and “SHARD+PB” (SHIELD, antigen retrieval, and photobleaching). These conditions are compared qualitatively and quantitatively in Figures 1–5.

### 3.6 Alkaline decolorization treatment

**Figure 6:**
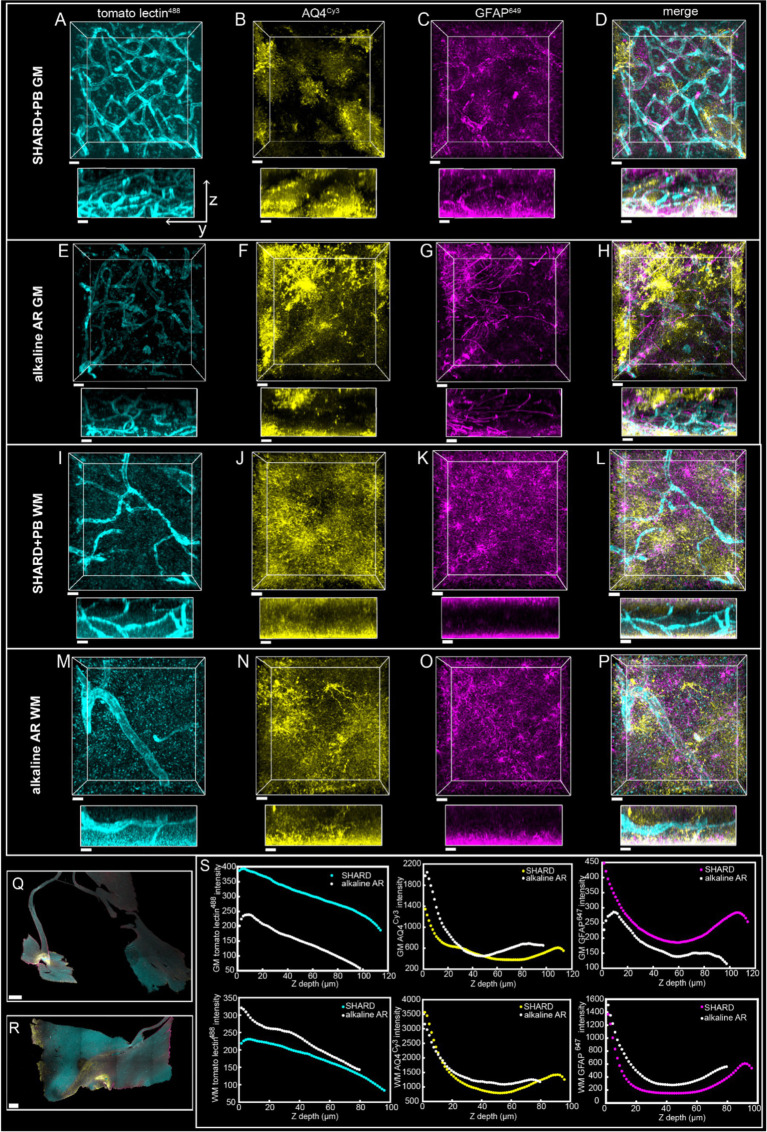
Qualitative Comparison of SHARD+PB vs. an alkaline antigen retrieval treatment. 3D renderings of immunofluorescent stains in the DLF of case u11 following treatment with the SHARD+PB condition **(A–D,I–L)** and the alkaline AR protocol **(E–H,M–P)** in the gray matter (**A–H**, 97.5 μm Z-stacks) and the white matter (**I–P**, 79.5 μm Z-stacks). The three stains shown are tomato lectin^488^ **(A,E,I,M)**, anti-AQ4^Cy3^ **(B,F,J,N)**, and anti-GFAP^647^ **(C,G,K,O)**. The overlay of all three is shown in **(D,H,L, P)**. All stains are shown as xy views (top) and yz views (bottom), both of which are raw data. Scale bar = 20 μm. **(Q)** Whole sample scans at 4x of u11 treated with the alkaline AR protocol **(Q)** and the SHARD+PB condition **(R)**, with a scale bar of 1 mm for both. **(S)** Plots of stain intensity vs. tissue Z depth in the gray matter (top row) and white matter (bottom row). Stain intensities in the tissue treated with alkaline antigen retrieval are plotted in white, and intensities in tissue treated with SHARD are plotted in the same colors in which they are displayed **(A–P)**.

A side-by-side comparison was completed on case u11 DLF tissue using the SHARD+PB condition and a previously published alkaline decolorization treatment (Scardigli et al., 2021). Briefly, before SHIELD processing, the section was treated with 30% (vol/vol) H_2_O_2_ diluted in deionized (DI) water for 1 h at RT and then washed three times in PBS for 10 min each. The sample was incubated in an alkaline antigen retrieval solution for 10 min at 95°C. The specimen was then cooled at RT for 30 min and washed in DI water for 5 min before incubating for 1 h in PBS. The section was immediately stained. This comparison is shown in Figure 6.

### 3.7 Staining

Sections were incubated for 2 h in a blocking solution of PBST with 5% normal donkey serum immediately before staining. Staining was conducted over two 24-h RT incubations with gentle shaking. For the sections used to compare different experimental conditions, each slice was incubated with a 10 μg anti-GFAP antibody cocktail (BioLegend, cat# 837602, San Diego, CA) and 12 μg anti-Aquaporin-4 (Abcam Ab 3,594, Boston, MA) in 1% normal donkey serum in PBST for a total volume of 1 mL. On the 2nd day, each slice was incubated with 40 μg tomato lectin^488^ (Vector Laboratories, Newark, CA), as previously described (Rosen et al., 2023), 6.7 μg Cy3-conjugated donkey anti-Rabbit secondary antibody, and 10 μg Alexa Fluor^®^488 (AF488)-conjugated donkey anti-mouse secondary (Jackson ImmunoResearch, 711–165-152 and 715–605-150, respectively, West Grove, PA) in PBST for a total volume of 1 mL. These immunofluorescence stains will be abbreviated as AQ4^Cy3^ and GFAP^647^.

**Figure 7:**
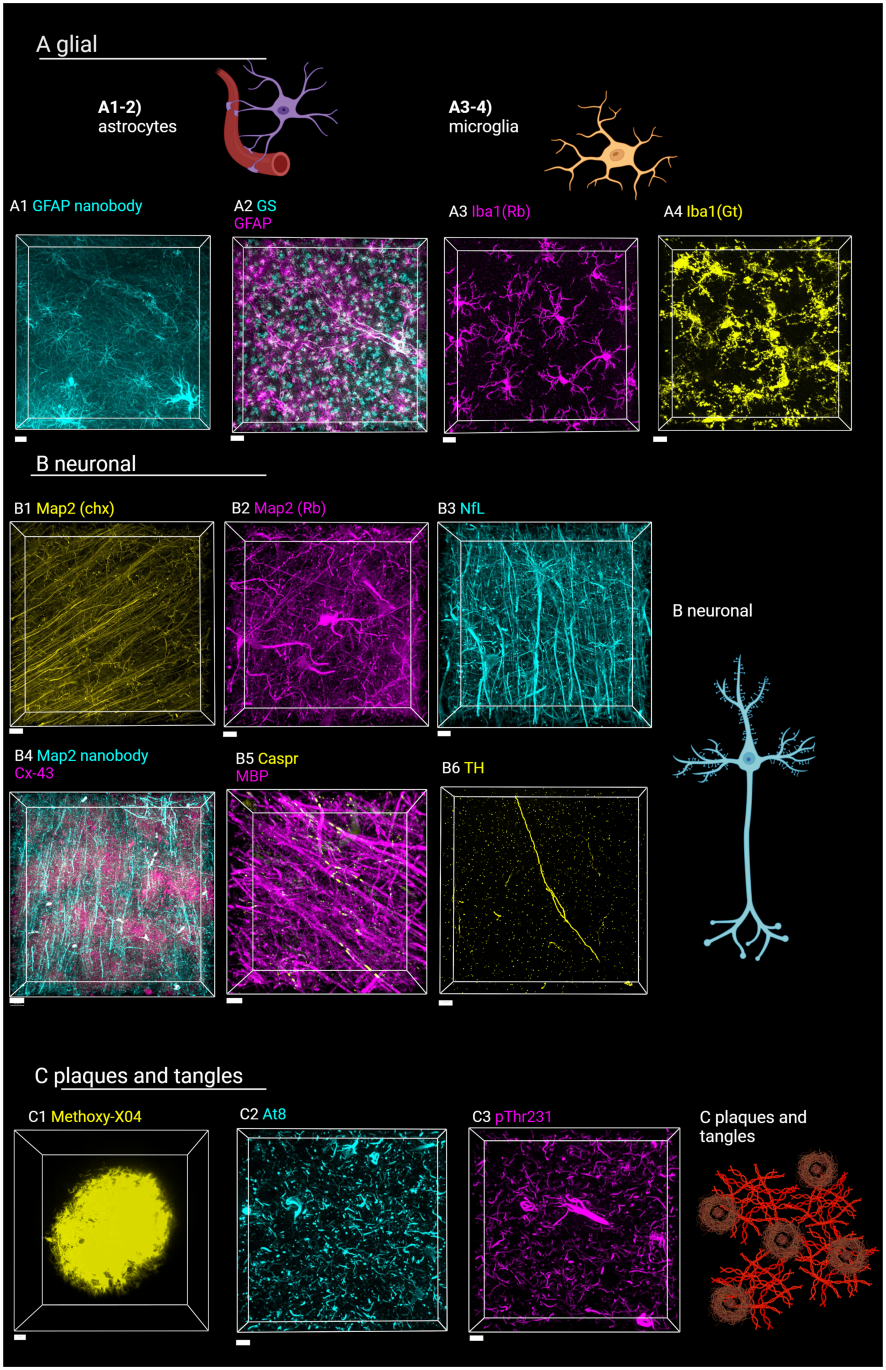
Staining of various targets of interest in the human brain with SHARD. Antibodies and stains are defined in Table 1. Images are taken in the DLF unless otherwise indicated. **(A)** Astrocytes and microglia. **(A1)** AF647-conjugated camelid anti-GFAP fluorescence in case u8, taken at 60x, 31.5 μm Z-stack, scale bar 10 μm. **(A2)** anti-GS^Cy3^ and anti-GFAP^647^ fluorescence in case u9 taken at 20x, 90 μm Z-stack, scale bar 30 μm. **(A3)** anti-Iba1^647^ (rabbit host species) fluorescence in case u10 taken at 60x, 45 μm Z-stack, scale bar 15 μm. **(A4)** Anti-Iba1^Cy3^ (goat host species) fluorescence in case u11 taken at 40x, 45 μm Z-stack, scale bar 15 μm. **(B)** Neuronal stains **(B1)** Anti-Map2^Cy3^ (chicken host species) fluorescence in case a3 parietal lobe taken at 40x, 10 μm scale bar, 94 μm Z-stack. **(B2)** Anti-Map2 (rabbit host species) fluorescence in case u13 taken at 60x, 68 μm Z-stack, 15 μm scale bar. **(B3)** Anti-neurofilament^647^ fluorescence in case u12 taken at 40x, 103 μm Z-stack, 20 μm scale bar **(B4)** anti-Map2^555^ nanobody and anti-Cx43^647^ fluorescence in case p4 taken at 40x, 103 μm Z-stack, 30 μm scale bar. **(B5)** Anti-MBP^647^ and anti-caspr^488^ fluorescence in case p5 entorhinal cortex taken at 60x, 48 μm Z-stack, 10 μm scale bar. **(B6)** Anti-TH^647^ fluorescence in case a1 taken at 40x, 53 μm Z-stack, 20 μm scale bar. **(C1)** Methoxy-X04 fluorescence in case u3, taken at 100x, 36 μm Z-stack, 5 μm scale bar. **(C2)** Anti-AT8^647^ fluorescence in case u9 taken at 20x, 34 μm Z-stack, 15 μm sale bar. **(C3)** Anti-pThr231^488^ fluorescence in case u9 taken at 20x, 46 μm Z-stack, 15 μm scale bar. Created in BioRender (Rosen, 2024), BioRender.com/f02b703.

Samples shown in Figure 7 were prepared with SHARD and stained with primary antibodies listed in Table 1. After 24 h of incubation with the primary antibody, sections were washed three times in PBST. Unless the stain was a small-molecule dye or conjugated primary, sections were incubated in the appropriate secondary antibody from Jackson ImmunoResearch such that the ratio of secondary to primary antibody was 2:1.

After the staining procedure, the samples were washed three times with PBST for 30 min each wash and incubated overnight in 4% PFA to post-fix the dye and antibodies to the tissue. The next day, the samples were washed three times, 2 h per wash, in PBS. Samples were incubated overnight in EasyIndex (LifeCanvas) before mounting onto a 25 × 75 × 1.0 mm glass slide (Fisher, Pittsburgh, PA) and covered with a No. 1.5, 22 × 30 mm glass coverslip (Corning GlassWorks, Corning, NY). EasyIndex (LifeCanvas, Cambridge, MA) was used for mounting media.

### 3.8 Imaging of cleared tissue

To quantify the three conditions, tissue slides were imaged on a Zeiss 880 confocal using a PLAN APO 20x/0.8 NA objective with Zen Black software (Zeiss, White Plains, NY). DyLight488, Cy3, and Alexa 647 were excited with 488 nm, 561 nm, and 633 nm wavelengths, respectively. Z-stacks of the available working distance were taken with optical sections spaced 1.5 μm apart, with a zoom of 1.8 and 4 line averaging. Pixel size was 0.115 × 0.115 × 1.5 μm and 16-bit. Pixel dwell was 2.05 μs. Emitted light was sent to the Airyscan detector, and 3D Airyscan processing was completed in Zen Black prior to image analysis. The pinhole radius was 266 μm for the tomato lectin^488^ channel, 449 μm for the Aq4^Cy3^ channel, and 598 μm for the GFAP^647^ channel. All image stacks were taken in the cortical gyri of the DLF, defined as the top third of the distance between the gyral crest and sulcus.

Images in Figure 7 were taken using either a Nikon Ti2 Eclipse Crest V2 spinning disc microscope or a Nikon AX-R microscope (Nikon, Melville, NY). The pinhole was set to 1 AU with a zoom size of 1.0 and pixel dwell time of 0.24 μs for all images acquired with the AX-R. The spinning disc has 70 μm pinholes. Nikon AX-R images were all taken with unidirectional scanning. Figures 7A1,A3,B2,B5 were taken with a CFI PLAN APO *λ* 60x/1.42 NA oil immersion objective with the Nikon spinning disc microscope. Figures 7A2,C2,C3 were taken with a CFI PLAN APO λ 20x/0.8 NA air objective on the Nikon spinning disc microscope. Figure 7C1 was taken with a CFI PLAN APO λ 100X/1.45 NA oil immersion objective with the Nikon spinning disc microscope. Figures 7A4,B1,B3,B6 were taken with a CFI PLAN APO 40x/1.25 NA silicone oil lens on the Nikon AX-R microscope using unidirectional scanning. Figures 7B4–5 was taken with a CFI PLAN APO λ 60x/1.42 NA oil immersion objective with the Nikon AX-R microscope.

**Figure 8:**
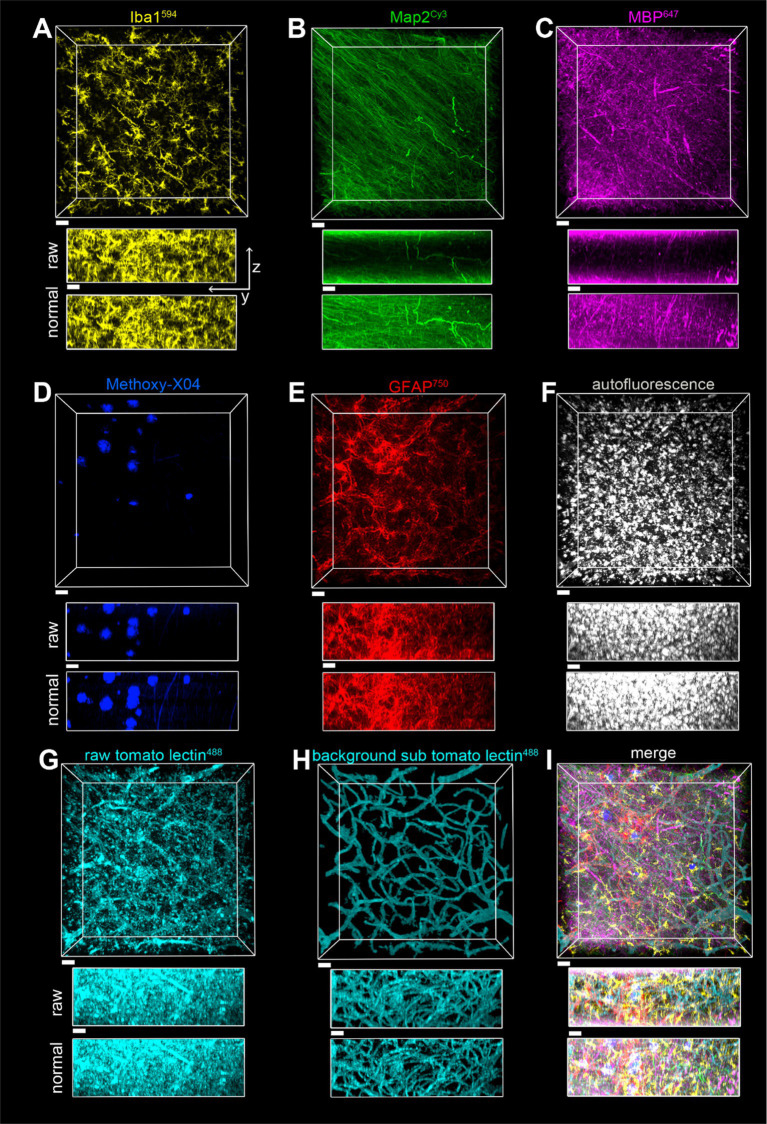
Imaging of a seven-color multiplex in the gray matter of the parietal lobe in case of a2, all in the same field of view. The scale bar is 30 μm for all. **(A)** Iba1^594^, **(B)** Map2^Cy3^, **(C)** MBP^647^, **(D)** Methoxy-X04, **(E)** GFAP^750^, **(F)** autofluorescence, **(G)** tomato lectin^488^, **(H)** tomato lectin^488^ after autofluorescence subtraction and image segmentation, **(I)** Overlay of stains shown in **(A–E,H)**. The z-stack is 148 μm, with a Z-step of 0.5 μm. yz view of image stacks is shown with both raw pixel intensities and pixel intensities after layer normalization preprocessing.

**Figure 9:**
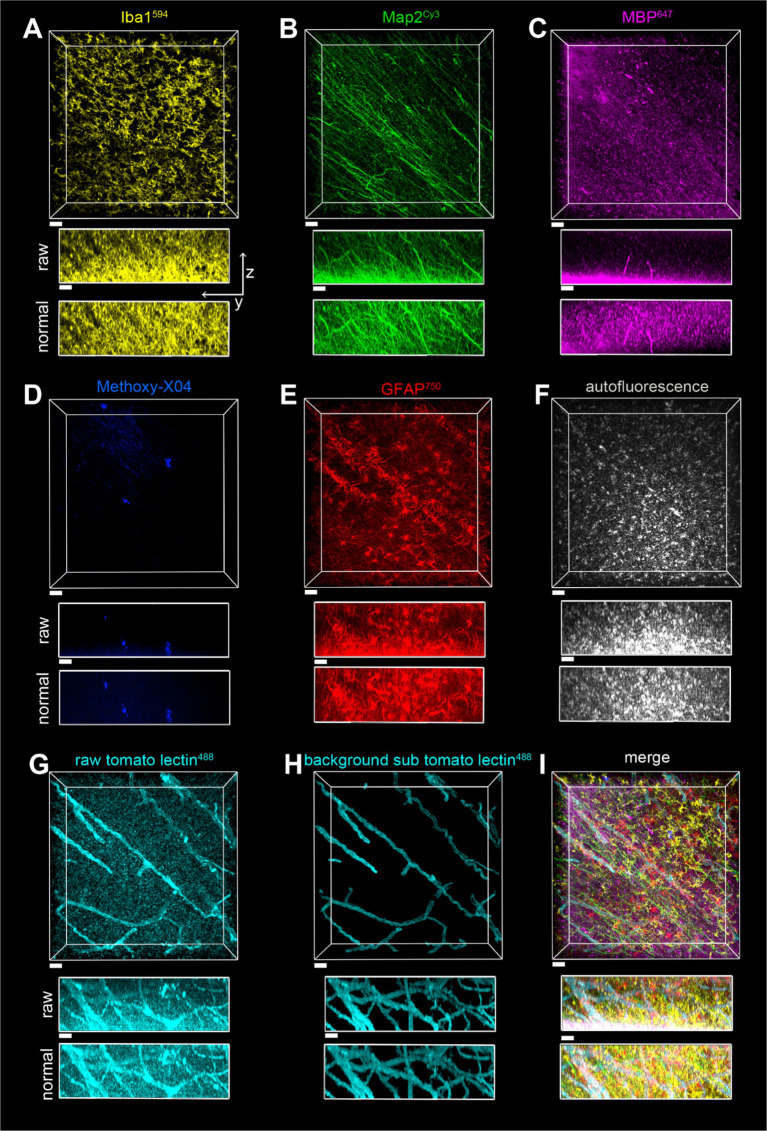
Imaging of a seven-color multiplex in the white matter of the parietal lobe in case of a2, all in the same field of view. The scale bar is 30 μm for all. **(A)** Iba1^594^, **(B)** Map2^Cy3^, **(C)** MBP^647^, **(D)** Methoxy-X04, **(E)** GFAP^750^, **(F)** autofluorescence, **(G)** tomato lectin^488^, **(H)** tomato lectin^488^ after autofluorescence subtraction and image segmentation, **(I)** Overlay of stains shown in **(A–E,H)**. The z-stack is 116 μm with a Z-step of 0.5 μm. yz view of image stacks is shown with both raw pixel intensities and pixel intensities after layer normalization preprocessing.

Images in Figures 8, 9 were acquired with a Nikon AX-R confocal microscope with two tunable gallium arsenide phosphide (GaAsP) detectors, a multi-alkaloid detector with emission filters, and a fixed GaAsP detector with emission filters. The pinhole was set to 1 AU for all channels with a zoom size of 1.0, unidirectional scanning, 4 line averaging, and pixel size of Parameters for the optical configurations used for the 7-color multiplex shown in Figures 8, 9 are displayed in Table 4. Imaging was done with a CFI PLAN APO 40x/1.25 NA silicone oil lens and unidirectional scanning. Data from the fluorophores was divided into two optical configurations: one with methoxy-X04, AlexaFluor488, autofluorescence, and AlexaFluor594, and the other with Cy3, AlexaFluor647, and AlexaFluor750. In this way, both optical configurations had two dyes that used both the tunable GaAsP detectors. Z-resolution was 0.4 μm. Images on a single plane were taken with both optical configurations before moving in Z onto the next plane.

**Table 4:** Nikon AX-R optical configuration parameters for immunofluorescent 7-plex in cleared postmortem human brain tissue.

Imaging target	Excitation wavelength (nm)	EMISSION RANGE (NM)	DETECTOR PARAMETERS
Methoxy-X04	405	418–475	MA R:447/57 T:490 LP
Tomato lectin^488^	488	510–544	tunable GaAsP
Map2^Cy3^	561	579–710	tunable GaAsP
Iba1^594^	594	615–636	tunable GaAsP
MBP^647^	633	664–710	tunable GaAsP
GFAP^750^	710	740–850	MA R: 740 LP T:728 SP
Autofluorescence	488	655–850	GaAsP R:655 LP T:650SP

Detectors listed are either multi-alkaloid (MA), tunable GaAsP, or non-tunable GaAsP with an emission filter.

A surface for the autofluorescence was created in Imaris 10 with segmentation based on pixel intensity and then thresholded to a volume of segmentation greater than 100 pixels. The intensity threshold was manually set to encompass all of the signals, as the purpose of this channel was to capture the low-intensity signals that appear in all channels. To subtract autofluorescence from the tomato lectin^488^ channel, pixels inside of the autofluorescent surface in the tomato lectin^488^ channel were set to zero, and the tomato lectin^488^ staining was also segmented based on pixel intensity and volume. Pixels outside of the resulting segmentation were set to zero, resulting in the background-subtracted tomato lectin^488^ images. The rationale for removing autofluorescence before segmentation is that in the fluorophore channels, there is no way to distinguish between autofluorescent pixel intensity and pixel intensity coming from fluorophores. It is easier to create an additional background subtracted channel and segment than to manually edit segmented surfaces individually to remove autofluorescence, which is a time-consuming process in Imaris. All images displayed are after a layer-normalizing preprocessing step that accounts for the effects of photobleaching.

### 3.9 Quantification

Segmentation was completed using Imaris (v10.0, Bitplane, Belfast, United Kingdom). Surface creation for each channel (GFAP^647^, AQ4^Cy3,^ and tomato lectin^488^) was based on background-subtracted pixel intensity and segment volume. Only these two parameters were used for the segmentation of GRAP^647^ and AQ4 ^Cy3^. For the tomato lectin^488^ channel, an additional filter was used for sphericity, removing sphere-shaped objects as blood vessels have tubular morphology. For each case, parameters for segmentation were created based on the SHARD+PB condition white matter and gray matter images, giving a total of six parameter sets (one gray matter, one white matter, for each channel). These parameters were then applied to the image sets from conditions 1 and 2 for all six channels, gray matter and white matter. The average pixel intensity in the created surface was the “signal” measurement used in the signal-to-noise ratio calculation. The average intensity of pixels outside of the surface was the “noise” intensity measurement. The signal-to-noise ratio (SNR) was calculated as the mean signal intensity divided by the mean noise intensity.

The fraction volume for each stain in each image stack was calculated as the total volume of the segmented surface divided by the total volume of the image stack. To account for any biological difference in fraction volume on a case-by-case basis, we normalized the fraction volume of the SHIELD only and SHARD conditions to the fraction volume of the SHARD+PB condition. Therefore, the normalized fraction volume of the SHARD+PB condition was always 1.

To quantify stain penetration depth and assess the utility of the normalized layer function in Imaris, we imported raw and normalized data into Arivis Pro for quantification. The mean pixel intensity was calculated for each plane in each of the raw and normalized channels. This was done for each of the 22 Z-stacks of each tissue treatment condition from 11 cases, as each case had two Z-stacks per condition, one in the white matter and one in the gray matter. For each condition and stain, standard errors were calculated for each Z position using the number of cases that had corresponding images at each Z position.

### 3.10 Statistical analyses

The fluorescence microscopy data were analyzed using SPSS (v. 27, IBM, Inc., Armonk, NY) and GraphPad Prism (v. 9.0.0, GraphPad Software, La Jolla, CA). An analysis of variance (ANOVA) was used to obtain means of signal intensity and SNR for tomato lectin, aquaporin, and GFAP stains in each experimental group. Because fraction volume was normalized based on the SHARD+PB condition, normalized fraction volume in the SHARD+PB condition had a standard error of zero and was not included in statistical comparisons. An unpaired t-test was used to compare the normalized fraction volume of the SHIELD-only condition versus the SHARD condition. Associations among measured image quality quantifiers were measured with linear regression models with age of death or PMI as the independent variable and signal intensity, noise intensity, normalized fraction volume, and SNR as the dependent variables. The significance level was set *a priori* to 5%.

## 4 Results

### 4.1 Antigen retrieval results in robust fluorescent microscopy images on cleared postmortem human brain tissue

SHIELD tissue treatment, passive delipidation, and staining were successful in visualizing blood vessels and astrocytes in three dimensions. Cases u1-7 and p1-4 were each subjected to SHIELD only, SHARD, and SHARD+PB conditions. Figure 1 shows tomato lectin^488^, AQ4^Cy3^, and GFAP^647^ fluorescence in the gray matter, and Figure 2 shows tomato lectin^488^, AQ4^Cy3^, and GFAP^647^ fluorescence in the white matter. Fluorescence was distinctive in the gray and white matter for all three stains. Tomato lectin^488^ fluorescence shows many branching curving vessels in the gray matter and more linear vessels in the white matter with fewer branches (Figures 1A,E,I compared to Figures 2A,E,I). Astrocyte density was generally greater in the white matter compared to the gray matter, as white matter showed more AQ4^Cy3^ and GFAP^647^ fluorescence density (Figures 2B,C,F,G,J,K) than the gray matter (Figures 2B,C,F,G,J,K). Staining quality appeared comparable among the three experimental conditions for tomato lectin^488^ (Figures 1, 2A vs. Figure 2E vs. Figure 2I) and AQ4^Cy3^ (Figures 1, 2B vs. Figure 2F vs. Figure 2J). However, the SHIELD-only condition produced minimal, if any, staining for GFAP^647^ (Figures 1, 2C) compared to the SHARD or SHARD+PB conditions (Figures 1, 2G,K).

Both photobleaching during confocal imaging and stain penetration into the tissue can be quantified by stain intensity vs. depth plots. Plots of raw data intensity vs. depth showed an initial decrease in staining intensity with increasing depth for all three stains (Figures 1, 2M,N, colored plots). The normalized layer function in Arivis, which standardizes the mean intensity of each channel it is applied to in each Z-slice, can be used to effectively eliminate the decrease of intensity through Z (Figures 1, 2M,N, white plots). Qualitatively, the normalized layer function creates uniformity of intensity in each Z slice, as seen in the yz views in Figures 1, 2A–L. Intensity vs. depth curves were U-shaped for AQ4^Cy3^, with planes at the ends of the Z-stack containing more intense pixels than the middle (Figures 1, 2M,N, middle yellow plots).

### 4.2 Antigen retrieval dramatically improved GFAP^647^ stain

Antigen retrieval significantly increased both mean signal brightness and mean detected volume of GFAP^647^ fluorescence in the DLF gray matter. In the gray matter, the mean segmented signal intensity was greater in the SHARD (1,000 ± 100) and SHARD+PB (900 ± 100) conditions than they were for the SHIELD only condition (300 ± 100), *p* = 0.0007 and 0.0019, respectively (Figure 3A). The mean normalized fraction volume was similarly higher in the SHARD condition (1.6 ± 0.4) compared to the SHIELD-only condition (0.02 ± 0.01), *p* < 0.0001 (Figure 3B). Only three cases had any GFAP^647^ detected by the same surface-building parameters applied to the SHARD+PB condition in the gray matter. The ratio of average pixel intensity inside the segmented surface vs. that outside the segmented surface (SNR) was 2.0 ± 0.8 in the SHIELD-only condition, 4.5 ± 0.8 in the SHARD condition, and 4.2 ± 0.7 in the SHARD+PB condition, none of which were significantly different (Figure 3C).

The same increases in signal brightness and normalized fraction volume of GFAP^647^ fluorescence were measured in the GFAP^647^ white matter. GFAP^647^ white matter signal intensity was significantly greater in the SHARD+PB condition (1,300 ± 200) compared to the SHIELD-only condition (130 ± 90, *p* = 0.0034) (Figure 3D). The GFAP^647^ white matter signal intensity in the SHARD condition (1,300 ± 300) was also significantly greater than that in the SHIELD-only condition (*p* = 0.0077). The normalized fraction volume of GFAP^647^ in the white matter was 1.7 ± 0.6, significantly greater than the equivalent in the SHIELD-only condition, 0.0004 ± 0.0009, *p* = 0.003 (Figure 3E). None of the cases showed any detected GFAP^647^ volume in the white matter in the SHIELD-only condition. Again, the mean SNR values of the three conditions were not significantly different, with values of 1.4 ± 0.8 for SHIELD only, 2.9 ± 0.4 for the SHARD condition, and 2.6 ± 0.3 for the SHARD+PB condition (Figure 3F).

### 4.3 Tomato lectin^488^ fluorescence was consistent across experimental conditions

There were no statistically significant differences in the gray matter for tomato lectin^488^ signal fluorescence intensity, normalized fraction volume, or SNR (Figures 4A–C). The white matter tomato lectin^488^ signal intensity in the SHARD condition (1,500 ± 200) was significantly greater than the signal intensity in the SHIELD-only condition (900 ± 100, *p* = 0.0021) and in the SHARD+PB condition (1,080 ± 60, *p* = 0.0207, Figure 4D). There were also no statistically significant differences in the white matter for tomato lectin^488^ normalized fraction volume, or SNR (Figures 4E,F).

### 4.4 AQ4^Cy3^ fluorescence was consistent across experimental conditions

There were no statistically significant differences in AQ4 ^Cy3^ signal intensity, normalized fraction volume, or SNR between the three conditions in the white matter or the gray matter (Figure 5). The gray and white matter AQ4 normalized fraction volume in the SHARD condition appears to be trending higher than in the SHIELD-only condition in both the gray and white matter, but this difference is not statistically significant (Figures 5B,E).

### 4.5 Alkaline antigen retrieval produced comparable image quality to SHARD but fragile tissue

High-quality immunofluorescence images were also produced using antigen retrieval in a pH nine solution and bleaching with hydrogen peroxide (Figures 6E–H,M). For direct comparison, a section from the same case was treated with the pH 6 SHARD+PB condition (Figures 6A–D,I). While most of the metrics of image quality were comparable, the alkaline antigen retrieval and peroxide bleaching were more effective at reducing background in the tomato lectin^488^ channel. The tomato lectin^488^ SNR of the SHARD+PB condition was 1.5 in the gray matter and 1.6 in the white matter. The tomato lectin^488^ SNR in the alkaline AR + peroxide bleach condition was higher at 3.2 in the gray matter and 2.4 in the white matter. The SHARD+PB condition detected about twice as much GFAP^647^ in the gray matter, with a fraction volume of 0.02 compared to 0.008 in the alkaline AR + peroxide bleach condition. While the alkaline AR + peroxide bleach condition did produce high-quality images, a major drawback to this technique was that the tissue was torn and brittle at the end of treatment (Figures 6Q,R). Penetration of all three stains was comparable regardless of SHARD or alkaline antigen retrieval treatment (Figure 6S).

### 4.6 3D visualization of additional markers with SHARD

We also tested 14 additional antibodies and one small-molecule dye for staining different cell types and pathology-associated markers with the antigen retrieval and tissue-clearing protocol (Table 1). The hope is to provide a reference by which future studies on human tissue may be completed. Images of these stains are shown in Figure 7, along with cartoons of the cell type or marker detected. Through these stains, astrocytes, microglia, neurons, plaques, and hyperphosphorylated tau tangles were successfully visualized.

Glial cells are a major target group we sought to detect. As described in sections 3.2 and 3.4, the GFAP and AQ4 antibodies detect astrocytes, with GFAP especially detecting the vast network of processes. As an alternative to the GFAP from BioLegend, a camelid GFAP nanobody conjugated to AlexaFluor647 dye also detects activated astrocytes (Figure 7A1). Nanobodies are engineered antibodies that are advantageous due to their small (~15 kDa) size (Sun et al., 2021). We further detected astrocytes with a glutamine synthetase antibody (Figure 7A2). Detecting microglia was successful with a well-established ionized calcium-binding adapter molecule I (Iba1) antibody from Wako (Figure 7A3). As another alternative, we also tested Wako’s Iba1 produced in goats (Figure 7A4).

Secondly, different types of neurons and neuronal components could be detected with the SHARD method. Microtubule-associated protein 2 (Map2) proved to be a successful target for visualizing neuronal cell bodies and processes, with a primary antibody produced in both chickens (Figure 7B1) and rabbits (Figure 7B2). Pan-neuronal staining is visualized with an anti-neurofilament antibody (Figure 7B3). As a direct staining alternative, an AlexaFluor555-conjugated Map2 primary nanobody also detected neuronal processes (Figure 7B4). A GeneTex myelin basic protein antibody was effective for visualizing the myelin sheath, and an Abcam antibody against the contactin-associated protein (caspr) highlights the ends of the axonal internodes (Figure 7B5). Intercellular gap junctions were visualized with an anti-connexin43 (Cx43) antibody (Figure 7B4). The anti-tyrosine hydroxylase (TH) antibody detects catecholaminergic principal neurons and processes (Figure 7B6).

Finally, we detected a few targets for neuropathological disease markers. Small-molecule dye Methoxy-X04 from Tocris Bioscience detected Aβ plaques, a hallmark of AD (Figure 7C1). Tangles of hyperphosphorylated tau (ptau) are also present in AD and in the pathognomonic lesion of CTE. We detected two variants of ptau with a widely used AT8 antibody (Figure 7C2) and an anti-pThr231 antibody (Figure 7C3).

### 4.7 Highly multiplexed immunofluorescence in cleared tissue

The toolbox of various stains described, plus the SHARD method, allowed for the visualization of six fluorescently stained targets in addition to autofluorescence. Provided that each primary antibody is from a different host species, multiple targets can be stained simultaneously without the cross-reactivity of fluorescent secondary antibodies. A seven-color multiplex was imaged in both gray matter (Figure 8) and white matter (Figure 9). With the Nikon AX-R, customizable detectors, and six different laser lines, we imaged a multiplex constituting of Iba1^594^ (goat host species, Figures 8A, 9B), Map2^Cy3^ (chicken host species, Figures 8B, 9B), MBP^647^ (rabbit host species, Figures 8C, 9C), Methoxy-X04 (small-molecule dye, Figures 8D, 9D), GFAP^750^ (mouse host species, Figures 8E, 9E), autofluorescence (Figures 8F, 9F), and tomato lectin^488^ (small-molecule dye, Figures 8G,H, 9G,H). Figures 8G, 9G show the tomato lectin^488^ raw data. Background-subtracted tomato lectin^488^ images are shown in Figures 8H, 9H.

Fluorophores were strategically paired with their targets. Because of the multitude of dyes in this multiplex, the Cy3 channel has the smallest range of detection without risking cross-detection from the AlexaFluor594 channel. For this reason, a robust antibody such as Map2 best suits this dye in the multiplex. From our experience, myelin is autofluorescent in red, and thus, we chose the AlexaFluor647 dye for labeling MBP. Similarly, autofluorescence of blood vessels is excited with a 488 nm light source, so tomato lectin^488^ was chosen to stain blood vessels. With these two strategic dye placements, nontarget autofluorescence was minimized across channels. Finally, autofluorescence in postmortem human tissue is usually strongest when excited by the 488 laser. Another reason that the DyLight 488 fluorophore was chosen to stain blood vessels is that blood vessels have obvious morphology that can be easily segmented.

A separate channel was also designated for autofluorescence. In our experience, autofluorescence can have a large Stokes shift. Because of this, we were able to excite autofluorescence with the 488 laser and use the same detector range as the AlexaFluor647 channel. After segmentation, pixels designated as autofluorescence were subtracted from the tomato lectin488 channel (see Figure 8G vs. Figure 8H and Figure 9G vs. Figure 9H). This autofluorescence can be segmented and subtracted off of any imaged channel to remove background.

### 4.8 Effectiveness of antigen retrieval persists across brain banks and AD status

There were no differences in the means of variables measured in cases without neurodegenerative disease compared to cases with neurodegenerative disease. Of the 11 cases quantified in the three conditions, four cases had AD. The means of signal intensities, signal-to-noise ratios, noise intensities, and normalized fraction volumes did not differ for tomato lectin, AQ4, or GFAP^647^ staining in the gray or white matter (Supplementary Table S1). There were also no significant differences when cases were grouped by Brain Bank of donation (NPBB vs. UNITE, Supplementary Table S2).

### 4.9 Effects of age and postmortem interval

Fluorescent noise intensity decreased with increasing age with tomato lectin, GFAP^647^, and AQ4 ^Cy3^ stains. This negative correlation was present in all three experimental conditions for all stains that had signal (see Table 5 for details).

**Table 5:** Linear regressions of the age of death vs. measured variables in the gray matter (first two columns) and the white matter (last two columns).

	Age at death GM regressions		Age at death WM regressions	
	β	*p*-value	β	*p*-value
SHIELD only tomato lectin noise intensity	−0.766	0.006	−0.766	0.006
SHARD tomato lectin noise intensity	−0.769	0.006	−0.769	0.006
SHARD+PB tomato lectin noise intensity	−0.767	0.006	−0.766	0.006
SHARD GFAP noise intensity	−0.758	0.007	−0.765	0.006
SHARD+PB GFAP noise intensity	−0.764	0.006	−0.765	0.006
SHIELD only AQ4 noise intensity	−0.755	0.007	−0.768	0.006
SHARD AQ4 noise intensity	−0.767	0.006	−0.767	0.006
SHARD+PB AQ4 noise intensity	−0.767	0.006	−0.767	0.006

There were a few increases in astrocytic staining measurements that correlated with increased PMI (Table 6). The white matter AQ4^Cy3^ normalized fraction volume showed a significant correlation with PMI. The SNR of tomato lectin^488^ fluorescence in the white matter increased significantly with PMI in the SHARD+PB condition (standardized *β* = 0.793, *p* = 0.004). The normalized white matter AQ4^Cy3^ fraction volume increased with PMI in the SHIELD-only condition (standardized *β* = 0.787, *p* = 0.004) and the SHARD condition (standardized *β* = 0.783, *p* = 0.004). There were no correlations of any measured quantification of image quality with the year the sample was received.

**Table 6:** Linear regressions of PMI vs. measured variables in the gray matter (first two columns and the white matter, last two columns).

	PMI GM regressions		PMI WM regressions	
	β	*p*-value	β	*p*-value
SHIELD only AQ4 normalized fraction volume	0.104	0.761	0.787	0.004
SHARD AQ4 normalized fraction volume	0.548	0.081	0.783	0.004

## 5 Discussion

We quantified the effectiveness of the SHARD method for robust immunostaining of cleared, long-term formaldehyde-fixed human postmortem brain tissue using tomato lectin^488^, AQ4^Cy3^, and GFAP^647^ stains. We compared immunostaining across delipidated tissues treated with SHIELD, SHARD, and SHARD followed by photobleaching with white light (SHARD+PB).

Acidic antigen retrieval significantly enhanced staining with the cytoskeletal GFAP antibody, as indicated by higher mean segmented signal intensity and a greater mean fraction of the image stack exhibiting the detected signal. Alkaline antigen retrieval produced comparable images, though acidic treatment had the advantage of preserving tissue integrity.

Both antigen retrieval and tissue clearing were effective across the three quantified markers and the three brain banks from which the samples were sourced, including those with AD. Additionally, we visualized a broader panel of antibodies compatible with the citrate antigen retrieval method. Notably, increased age at death correlated negatively with image noise across all channels.

Antigen retrieval with citrate dramatically increased the staining effectiveness of an intracellular marker. Without antigen retrieval, the GFAP^647^ signal was virtually absent. Heating in acidic conditions breaks formaldehyde crosslinking, which is crucial for unmasking binding sites, particularly cellular components like GFAP. In contrast, AQ4^Cy3^ and tomato lectin^488^ stains were effective not only in the SHARD conditions but also in the SHIELD-only condition. Tomato lectin likely does not require antigen retrieval due to its small-molecule structure, which experiences less steric hindrance when binding to its target, even in the presence of formaldehyde crosslinking. On the other hand, AQ4 is a cell membrane-bound target. Since SDS-based clearing dissolves cellular membranes, our findings suggest that SDS treatment alone is sufficient to expose the AQ4 antigen.

SHARD is equal to or more widely applicable than the few previously published studies combining antigen retrieval and tissue clearing. We found that a previously published method using alkaline antigen retrieval and hydrogen peroxide bleach (Scardigli et al., 2021) worked with our samples for microscopic regions but resulted in torn tissue sections. SHARD uses 10 mM citrate buffer, an order of magnitude lower than the citrate buffer used in more recent work in the field (Woelfle et al., 2023) but in line with methods documented a few decades ago (Jiao et al., 1999; Brorson, 2002; Ino, 2003; Namimatsu et al., 2005; Shi et al., 2007). The advantage of the method described here is that it is compatible with long-term, immersion-fixed human brain tissue stored in formaldehyde, and the treatment results are intact tissue sections. Our analyses show that this method works well despite any unavoidable variability of using human tissue, such as neurodegenerative disease status, varying age of death, and postmortem interval.

Intensity vs. depth analysis revealed uneven tissue penetration with SHARD. Because of confocal imaging, it is impossible to conclude that inhomogeneity in average pixel intensity throughout Z is entirely due to differential stain diffusion throughout the tissue. However, the U-shaped curves seen with AQ4^Cy3^ staining intensity would only be seen if there was a higher concentration of the stain at the edges of the sample. GFAP^647^ also sometimes displayed a U-shaped curve (Figure 6S), indicating that the edges of the sample were exposed to a higher concentration of the stain. The relatively uniform yz views of tomato lectin^488^ staining raw data would indicate that most of the decrease in staining intensity vs. imaging depth is due to photobleaching. A small-molecule dye would likely achieve more homogenous diffusion throughout samples than immunofluorescent antibodies. Even though samples were 200-μm thick, the acquired Z-stacks were closer to 100 μm due to both the sample not being flat and sample compression upon mounting. Further optimization is needed to achieve immunofluorescent staining homogeneity, but in the meantime, a computational correction such as the normalized layers function in Imaris results in uniformity of pixel intensities throughout a Z-stack (see Figures 1, 2M,N and yz views in Figures 1, 2A–L, 6A–P, 7, 8). As the normalized layer function leads to higher noise at deeper stacks, staining homogeneity through Z would be most advantageous. The use of expansion microscopy, nanobodies, fluorescently conjugated primary antibodies, and/or small-molecule dyes to stain tissue would also lead to more uniform staining.

Acidic antigen retrieval worked well across various conditions, including PMI, duration of storage in fixative, and age. We show data and images from samples with PMIs from 10 to 72 h and with ages of death ranging from 35 to 93 years. PMI correlated positively with normalized AQ4^Cy3^ fraction volume in the white matter. This is likely due to the breakdown of the membranes prior to clearing, causing AQ4 to spread beyond localized astrocytic cell bodies in fresher tissue. Interestingly, age at death had a negative correlation with noise intensity in all three quantified stains in both the gray and white matter. This was the only correlation that was universally true regardless of tissue treatment and stain and is likely due to the decrease in total brain lipids after the first 40–50 years of life (Naudí et al., 2015).

SHARD is highly suited for high multiplexing, allowing up to seven stains to be visualized simultaneously, provided that attention is given to the primary antibodies’ host species and the emission spectra of the detected stains. For optimal noise reduction, aligning the optical parameters of a target’s autofluorescence with the fluorophore used for immunofluorescence visualization is highly advantageous. For example, based on our experience, myelin exhibits autofluorescent when excited by a 633 nm laser, making myelin basic protein an ideal candidate for the AlexaFluor^647^ dye. In this way, the myelin autofluorescence from the 633 nm laser will overlap with the AlexaFluor^647^ fluorescence intensity at the target when bound to a secondary antibody for MBP. However, if AlexaFluor^647^ is used to stain a different target, there may still be autofluorescence corresponding to myelin morphology.

Non-specific background can also be significantly reduced by imaging an additional channel configured specifically for autofluorescence and subtracting that channel’s segmentation from the immunofluorescent channels during image analysis. In our 7-plex experiment, autofluorescence was subtracted from the tomato lectin^488^ channel. The distinct morphology of blood vessels makes them an ideal target for the AlexaFluor^488^ channel, which exhibits the highest spectral autofluorescence.

Photobleaching as a background-reducing step had less of an effect than expected, based on previous studies (Duong and Han, 2013; Ku et al., 2020). The photobleaching protocol was believed to eliminate autofluorescence so that the SHARD+PB signals would consist purely of immunofluorescence without autofluorescence.

However, signal and noise measurements were not statistically different between the SHARD and SHARD+PB conditions for GFAP^647^, AQ4^Cy3^, and gray matter tomato lectin^488^. Interestingly, white matter tomato lectin^488^ signal intensity was significantly lower in the SHARD+PB condition compared to the SHARD condition. This decrease suggests that significant noise may have contributed to the tomato lectin^488^ signal in the SHARD condition. Despite this, the SNR of white matter tomato lectin^488^ was not significantly affected by photobleaching, indicating that photobleaching likely did not impact overall image quality. Instead, creating a separate channel for autofluorescence proved more effective than photobleaching for removing non-specific fluorescent signals.

The limitations of this study include the small sample size and the fact that only male donors were used for comparison between tissue treatment conditions. The diversity of the study population was constrained by the availability of tissue from the UNITE, NPBB, and ADRC brain banks. Future studies aimed at refining tissue treatment methods should include donors of different genders to increase generalizability. Despite the small sample size, to the best of our knowledge, this is the largest systematically quantified comparison of different tissue treatment techniques for 3D microscopy. This study is not intended to serve as an endpoint in SHIELD tissue clearing optimization; rather, it represents a waypoint for further advancements. Further development could explore the use of thicker tissue sections, employing electrophoresis-driven clearing and staining, multi-photon or light sheet microscopy, and potentially higher multiplexing through fluorescence lifetime imaging (FLIM).

Overall, the SHARD method paves the way for new areas of research using optically cleared postmortem human brain tissue that has been preserved long-term. The combination of antigen retrieval and tissue clearing provides a powerful approach for visualizing targets that might otherwise be undetectable. The markers highlighted in this study can be utilized to investigate human disease through microscopy, with potential applications in a variety of research contexts.

## Data Availability

The raw data supporting the conclusions of this article will be made available by the authors, without undue reservation.
