# Supplementary material for: SHARD: an improved method for staining and visualizing multiplex immunofluorescence in optically cleared postmortem human brain tissue
Source: Front Neurosci. 2024 Oct 9;18:1474617. doi: 10.3389/fnins.2024.1474617 (PMC11496292; doi:10.3389/fnins.2024.1474617)
Supplement: Supplementary file 1 [file Table_1.docx]

**Supplemental**

**Table S1** Comparison of means of measured variables between AD and non-AD groups in the gray matter “GM” and white matter “WM”

|  | | Sum of Squares | Sig. |  | | Sum of Squares | Sig. |
| --- | --- | --- | --- | --- | --- | --- | --- |
| SHIELD only tomato lectin GM SNR | Between Groups | 8.525 | 0.435 | SHIELD only tomato lectin WM SNR | Between Groups | 0.086 | 0.704 |
|  | Within Groups | 115.015 |  |  | Within Groups | 5.020 |  |
|  | Total | 123.540 |  |  | Total | 5.106 |  |
| SHARD tomato lectin GM SNR | Between Groups | 0.193 | 0.428 | SHARD tomato lectin WM SNR | Between Groups | 1.104 | 0.249 |
|  | Within Groups | 2.520 |  |  | Within Groups | 6.542 |  |
|  | Total | 2.713 |  |  | Total | 7.645 |  |
| SHARD+PB tomato lectin GM SNR | Between Groups | 34.205 | 0.446 | SHARD+PB tomato lectin WM SNR | Between Groups | 0.639 | 0.124 |
|  | Within Groups | 485.420 |  |  | Within Groups | 1.995 |  |
|  | Total | 519.625 |  |  | Total | 2.634 |  |
| SHIELD only GFAP GM SNR | Between Groups | 4.631 | 0.458 | SHIELD only GFAP WM SNR | Between Groups | 0.071 | 0.928 |
|  | Within Groups | 69.436 |  |  | Within Groups | 74.015 |  |
|  | Total | 74.067 |  |  | Total | 74.086 |  |
| SHARD GFAP GM SNR | Between Groups | 6.122 | 0.351 | SHARD GFAP WM SNR | Between Groups | 0.486 | 0.586 |
|  | Within Groups | 56.943 |  |  | Within Groups | 13.670 |  |
|  | Total | 63.065 |  |  | Total | 14.155 |  |
| SHARD+PB GFAP GM SNR | Between Groups | 0.014 | 0.962 | SHARD+PB GFAP WM SNR | Between Groups | 0.148 | 0.722 |
|  | Within Groups | 49.992 |  |  | Within Groups | 9.889 |  |
|  | Total | 50.006 |  |  | Total | 10.037 |  |
| SHIELD only AQ4 GM SNR | Between Groups | 0.945 | 0.356 | SHIELD only AQ4 WM SNR | Between Groups | 0.027 | 0.870 |
|  | Within Groups | 8.982 |  |  | Within Groups | 8.711 |  |
|  | Total | 9.927 |  |  | Total | 8.738 |  |
| SHARD AQ4 GM SNR | Between Groups | 0.020 | 0.827 | SHARD AQ4 WM SNR | Between Groups | 1.345 | 0.308 |
|  | Within Groups | 3.522 |  |  | Within Groups | 10.390 |  |
|  | Total | 3.541 |  |  | Total | 11.735 |  |
| SHARD+PB AQ4 GM SNR | Between Groups | 0.054 | 0.789 | SHARD+PB AQ4 WM SNR | Between Groups | 0.569 | 0.258 |
|  | Within Groups | 6.455 |  |  | Within Groups | 3.516 |  |
|  | Total | 6.510 |  |  | Total | 4.085 |  |
| SHIELD only GM tomato lectin signal intensity | Between Groups | 685646.182 | 0.444 | SHIELD only WM tomato lectin signal intensity | Between Groups | 121009.821 | 0.305 |
|  | Within Groups | 9639700.000 |  |  | Within Groups | 918900.179 |  |
|  | Total | 10325346.182 |  |  | Total | 1039910.000 |  |
| SHARD GM tomato lectin signal intensity | Between Groups | 278882.545 | 0.319 | SHARD WM tomato lectin signal intensity | Between Groups | 6014.029 | 0.894 |
|  | Within Groups | 2259350.000 |  |  | Within Groups | 2894925.607 |  |
|  | Total | 2538232.545 |  |  | Total | 2900939.636 |  |
| SHARD+PB GM tomato lectin signal intensity | Between Groups | 949383.117 | 0.026 | SHARD+PB WM tomato lectin signal intensity | Between Groups | 108882.081 | 0.108 |
|  | Within Groups | 1208753.429 |  |  | Within Groups | 308092.464 |  |
|  | Total | 2158136.545 |  |  | Total | 416974.545 |  |
| SHIELD only GFAP GM signal intensity | Between Groups | 18159.821 | 0.739 | SHIELD only GFAP WM signal intensity | Between Groups | 17475.325 | 0.675 |
|  | Within Groups | 1381540.179 |  |  | Within Groups | 835926.857 |  |
|  | Total | 1399700.000 |  |  | Total | 853402.182 |  |
| SHARD GFAP GM signal intensity | Between Groups | 4785.052 | 0.862 | SHARD GFAP WM signal intensity | Between Groups | 1416993.120 | 0.259 |
|  | Within Groups | 1343419.857 |  |  | Within Groups | 8770035.607 |  |
|  | Total | 1348204.909 |  |  | Total | 10187028.727 |  |
| SHARD+PB GFAP GM signal intensity | Between Groups | 31483.753 | 0.691 | SHARD+PB GFAP WM signal intensity | Between Groups | 1127115.013 | 0.228 |
|  | Within Groups | 1680280.429 |  |  | Within Groups | 6068739.714 |  |
|  | Total | 1711764.182 |  |  | Total | 7195854.727 |  |
| SHIELD only AQ4 GM signal intensity | Between Groups | 63085.922 | 0.454 | SHIELD only AQ4 WM signal intensity | Between Groups | 68581.870 | 0.265 |
|  | Within Groups | 928811.714 |  |  | Within Groups | 436056.857 |  |
|  | Total | 991897.636 |  |  | Total | 504638.727 |  |
| SHARD AQ4 GM signal intensity | Between Groups | 2457.468 | 0.749 | SHARD AQ4 WM signal intensity | Between Groups | 139336.367 | 0.166 |
|  | Within Groups | 203446.714 |  |  | Within Groups | 551296.179 |  |
|  | Total | 205904.182 |  |  | Total | 690632.545 |  |
| SHARD+PB AQ4 GM signal intensity | Between Groups | 198.081 | 0.940 | SHARD+PB AQ4 WM signal intensity | Between Groups | 176162.195 | 0.272 |
|  | Within Groups | 298844.464 |  |  | Within Groups | 1159674.714 |  |
|  | Total | 299042.545 |  |  | Total | 1335836.909 |  |
| SHIELD only tomato lectin GM noise intensity | Between Groups | 3343878545.271 | 0.484 | SHIELD only tomato lectin WM noise intensity | Between Groups | 89106618.094 | 0.474 |
|  | Within Groups | 56455530560.239 |  |  | Within Groups | 1438735856.465 |  |
|  | Total | 59799409105.509 |  |  | Total | 1527842474.559 |  |
| SHARD tomato lectin GM noise intensity | Between Groups | 320327873.298 | 0.470 | SHARD tomato lectin WM noise intensity | Between Groups | 81155028.554 | 0.460 |
|  | Within Groups | 5063050320.265 |  |  | Within Groups | 1225920574.694 |  |
|  | Total | 5383378193.563 |  |  | Total | 1307075603.248 |  |
| SHARD+PB tomato lectin GM noise intensity | Between Groups | 2998532891.171 | 0.478 | SHARD+PB tomato lectin WM noise intensity | Between Groups | 250654408.943 | 0.480 |
|  | Within Groups | 49144986025.494 |  |  | Within Groups | 4151186465.169 |  |
|  | Total | 52143518916.664 |  |  | Total | 4401840874.112 |  |
| SHIELD only GFAP GM noise intensity | Between Groups | 30652.776 | 0.169 | SHIELD only GFAP WM noise intensity | Between Groups | 15986.004 | 0.408 |
|  | Within Groups | 123099.086 |  |  | Within Groups | 191027.935 |  |
|  | Total | 153751.862 |  |  | Total | 207013.939 |  |
| SHARD GFAP GM noise intensity | Between Groups | 20242255.513 | 0.483 | SHARD GFAP WM noise intensity | Between Groups | 926141830.097 | 0.472 |
|  | Within Groups | 340396252.323 |  |  | Within Groups | 14777655395.204 |  |
|  | Total | 360638507.836 |  |  | Total | 15703797225.301 |  |
| SHARD+PB GFAP GM noise intensity | Between Groups | 7709187.234 | 0.437 | SHARD+PB GFAP WM noise intensity | Between Groups | 2977722874.326 | 0.473 |
|  | Within Groups | 104745398.888 |  |  | Within Groups | 47808218755.053 |  |
|  | Total | 112454586.122 |  |  | Total | 50785941629.379 |  |
| SHIELD only AQ4 GM noise intensity | Between Groups | 3622771.744 | 0.524 | SHIELD only AQ4 WM noise intensity | Between Groups | 284148813.964 | 0.483 |
|  | Within Groups | 74299076.151 |  |  | Within Groups | 4783587744.796 |  |
|  | Total | 77921847.896 |  |  | Total | 5067736558.760 |  |
| SHARD AQ4 GM noise intensity | Between Groups | 781137847.868 | 0.479 | SHARD AQ4 WM noise intensity | Between Groups | 1879046709.065 | 0.480 |
|  | Within Groups | 12906356967.522 |  |  | Within Groups | 31110241136.600 |  |
|  | Total | 13687494815.390 |  |  | Total | 32989287845.666 |  |
| SHARD+PB AQ4 GM noise intensity | Between Groups | 171886499.184 | 0.478 | SHARD+PB AQ4 WM noise intensity | Between Groups | 892064796.545 | 0.478 |
|  | Within Groups | 2821575794.130 |  |  | Within Groups | 14627561065.179 |  |
|  | Total | 2993462293.313 |  |  | Total | 15519625861.725 |  |
| SHIELD only tomato lectin GM normalized fraction volume | Between Groups | 0.250 | 0.462 | SHIELD only tomato lectin WM normalized fraction volume | Between Groups | 0.393 | 0.243 |
|  | Within Groups | 3.815 |  |  | Within Groups | 2.265 |  |
|  | Total | 4.066 |  |  | Total | 2.658 |  |
| SHARD tomato lectin GM normalized fraction volume | Between Groups | 0.924 | 0.227 | SHARD tomato lectin WM normalized fraction volume | Between Groups | 0.090 | 0.271 |
|  | Within Groups | 4.938 |  |  | Within Groups | 0.591 |  |
|  | Total | 5.862 |  |  | Total | 0.682 |  |
| SHIELD only GM GFAP normalized fraction volume | Between Groups | 0.004 | 0.167 | SHIELD only WM GFAP normalized fraction volume | Between Groups | 0.000 | 0.098 |
|  | Within Groups | 0.015 |  |  | Within Groups | 0.000 |  |
|  | Total | 0.019 |  |  | Total | 0.000 |  |
| SHARD GM GFAP normalized fraction volume | Between Groups | 4.488 | 0.095 | SHARD WM GFAP normalized fraction volume | Between Groups | 30.432 | 0.652 |
|  | Within Groups | 11.604 |  |  | Within Groups | 1262.101 |  |
|  | Total | 16.092 |  |  | Total | 1292.533 |  |
| SHIELD only AQ4 GM normalized fraction volume | Between Groups | 3.533 | 0.130 | SHIELD only AQ4 WM normalized fraction volume | Between Groups | 9.152 | 0.275 |
|  | Within Groups | 11.432 |  |  | Within Groups | 61.059 |  |
|  | Total | 14.965 |  |  | Total | 70.211 |  |
| SHARD AQ4 GM normalized fraction volume | Between Groups | 8.737 | 0.083 | SHARD AQ4 WM normalized fraction volume | Between Groups | 9.549 | 0.320 |
|  | Within Groups | 20.747 |  |  | Within Groups | 77.429 |  |
|  | Total | 29.484 |  |  | Total | 86.978 |  |

**Table S2** Comparison of means of measured variables between groups originating in UNITE and NPBB Brain Bank in the gray matter “GM” and white matter “WM”

|  | | Sum of Squares | Sig. |  | | Sum of Squares | Sig. |
| --- | --- | --- | --- | --- | --- | --- | --- |
| SHIELD only tomato lectin GM SNR | Between Groups | 15.131 | 0.291 | SHIELD only tomato lectin WM SNR | Between Groups | 0.472 | 0.363 |
|  | Within Groups | 108.409 |  |  | Within Groups | 4.634 |  |
|  | Total | 123.540 |  |  | Total | 5.106 |  |
| SHARD tomato lectin GM SNR | Between Groups | 0.231 | 0.384 | SHARD tomato lectin WM SNR | Between Groups | 0.701 | 0.365 |
|  | Within Groups | 2.483 |  |  | Within Groups | 6.944 |  |
|  | Total | 2.713 |  |  | Total | 7.645 |  |
| SHARD+PB tomato lectin GM SNR | Between Groups | 103.949 | 0.168 | SHARD+PB tomato lectin WM SNR | Between Groups | 0.130 | 0.512 |
|  | Within Groups | 415.676 |  |  | Within Groups | 2.504 |  |
|  | Total | 519.625 |  |  | Total | 2.634 |  |
| SHIELD only GFAP GM SNR | Between Groups | 1.810 | 0.646 | SHIELD only GFAP WM SNR | Between Groups | 6.277 | 0.385 |
|  | Within Groups | 72.257 |  |  | Within Groups | 67.809 |  |
|  | Total | 74.067 |  |  | Total | 74.086 |  |
| SHARD GFAP GM SNR | Between Groups | 1.439 | 0.658 | SHARD GFAP WM SNR | Between Groups | 4.312 | 0.078 |
|  | Within Groups | 61.627 |  |  | Within Groups | 9.844 |  |
|  | Total | 63.065 |  |  | Total | 14.155 |  |
| SHARD+PB GFAP GM SNR | Between Groups | 0.506 | 0.769 | SHARD+PB GFAP WM SNR | Between Groups | 0.599 | 0.469 |
|  | Within Groups | 49.500 |  |  | Within Groups | 9.438 |  |
|  | Total | 50.006 |  |  | Total | 10.037 |  |
| SHIELD only AQ4 GM SNR | Between Groups | 0.027 | 0.878 | SHIELD only AQ4 WM SNR | Between Groups | 0.119 | 0.733 |
|  | Within Groups | 9.900 |  |  | Within Groups | 8.619 |  |
|  | Total | 9.927 |  |  | Total | 8.738 |  |
| SHARD AQ4 GM SNR | Between Groups | 0.158 | 0.533 | SHARD AQ4 WM SNR | Between Groups | 2.864 | 0.122 |
|  | Within Groups | 3.384 |  |  | Within Groups | 8.871 |  |
|  | Total | 3.541 |  |  | Total | 11.735 |  |
| SHARD+PB AQ4 GM SNR | Between Groups | 0.064 | 0.771 | SHARD+PB AQ4 WM SNR | Between Groups | 0.182 | 0.533 |
|  | Within Groups | 6.445 |  |  | Within Groups | 3.904 |  |
|  | Total | 6.510 |  |  | Total | 4.085 |  |
| SHIELD only GM tomato lectin signal intensity | Between Groups | 964544.468 | 0.361 | SHIELD only WM tomato lectin signal intensity | Between Groups | 43041.821 | 0.549 |
|  | Within Groups | 9360801.714 |  |  | Within Groups | 996868.179 |  |
|  | Total | 10325346.182 |  |  | Total | 1039910.000 |  |
| SHARD GM tomato lectin signal intensity | Between Groups | 873885.831 | 0.058 | SHARD WM tomato lectin signal intensity | Between Groups | 736570.922 | 0.114 |
|  | Within Groups | 1664346.714 |  |  | Within Groups | 2164368.714 |  |
|  | Total | 2538232.545 |  |  | Total | 2900939.636 |  |
| SHARD+PB GM tomato lectin signal intensity | Between Groups | 466286.545 | 0.150 | SHARD+PB WM tomato lectin signal intensity | Between Groups | 77636.688 | 0.185 |
|  | Within Groups | 1691850.000 |  |  | Within Groups | 339337.857 |  |
|  | Total | 2158136.545 |  |  | Total | 416974.545 |  |
| SHIELD only GFAP GM signal intensity | Between Groups | 13156.393 | 0.777 | SHIELD only GFAP WM signal intensity | Between Groups | 13143.325 | 0.716 |
|  | Within Groups | 1386543.607 |  |  | Within Groups | 840258.857 |  |
|  | Total | 1399700.000 |  |  | Total | 853402.182 |  |
| SHARD GFAP GM signal intensity | Between Groups | 124242.195 | 0.364 | SHARD GFAP WM signal intensity | Between Groups | 450776.263 | 0.535 |
|  | Within Groups | 1223962.714 |  |  | Within Groups | 9736252.464 |  |
|  | Total | 1348204.909 |  |  | Total | 10187028.727 |  |
| SHARD+PB GFAP GM signal intensity | Between Groups | 19552.325 | 0.754 | SHARD+PB GFAP WM signal intensity | Between Groups | 523009.299 | 0.423 |
|  | Within Groups | 1692211.857 |  |  | Within Groups | 6672845.429 |  |
|  | Total | 1711764.182 |  |  | Total | 7195854.727 |  |
| SHIELD only AQ4 GM signal intensity | Between Groups | 20114.029 | 0.676 | SHIELD only AQ4 WM signal intensity | Between Groups | 30620.263 | 0.465 |
|  | Within Groups | 971783.607 |  |  | Within Groups | 474018.464 |  |
|  | Total | 991897.636 |  |  | Total | 504638.727 |  |
| SHARD AQ4 GM signal intensity | Between Groups | 20227.325 | 0.348 | SHARD AQ4 WM signal intensity | Between Groups | 110126.545 | 0.224 |
|  | Within Groups | 185676.857 |  |  | Within Groups | 580506.000 |  |
|  | Total | 205904.182 |  |  | Total | 690632.545 |  |
| SHARD+PB AQ4 GM signal intensity | Between Groups | 5520.831 | 0.690 | SHARD+PB AQ4 WM signal intensity | Between Groups | 94080.159 | 0.430 |
|  | Within Groups | 293521.714 |  |  | Within Groups | 1241756.750 |  |
|  | Total | 299042.545 |  |  | Total | 1335836.909 |  |
| SHIELD only tomato lectin GM noise intensity | Between Groups | 3472010058.721 | 0.475 | SHIELD only tomato lectin WM noise intensity | Between Groups | 86615253.158 | 0.481 |
|  | Within Groups | 56327399046.788 |  |  | Within Groups | 1441227221.401 |  |
|  | Total | 59799409105.509 |  |  | Total | 1527842474.559 |  |
| SHARD tomato lectin GM noise intensity | Between Groups | 291382014.427 | 0.491 | SHARD tomato lectin WM noise intensity | Between Groups | 65185919.574 | 0.509 |
|  | Within Groups | 5091996179.136 |  |  | Within Groups | 1241889683.674 |  |
|  | Total | 5383378193.563 |  |  | Total | 1307075603.248 |  |
| SHARD+PB tomato lectin GM noise intensity | Between Groups | 2978353819.551 | 0.479 | SHARD+PB tomato lectin WM noise intensity | Between Groups | 253324458.314 | 0.477 |
|  | Within Groups | 49165165097.114 |  |  | Within Groups | 4148516415.798 |  |
|  | Total | 52143518916.664 |  |  | Total | 4401840874.112 |  |
| SHIELD only GFAP GM noise intensity | Between Groups | 4250.455 | 0.625 | SHIELD only GFAP WM noise intensity | Between Groups | 109.328 | 0.947 |
|  | Within Groups | 149501.407 |  |  | Within Groups | 206904.611 |  |
|  | Total | 153751.862 |  |  | Total | 207013.939 |  |
| SHARD GFAP GM noise intensity | Between Groups | 20273115.797 | 0.483 | SHARD GFAP WM noise intensity | Between Groups | 882812743.729 | 0.483 |
|  | Within Groups | 340365392.039 |  |  | Within Groups | 14820984481.572 |  |
|  | Total | 360638507.836 |  |  | Total | 15703797225.301 |  |
| SHARD+PB GFAP GM noise intensity | Between Groups | 6267778.076 | 0.485 | SHARD+PB GFAP WM noise intensity | Between Groups | 2924727214.359 | 0.477 |
|  | Within Groups | 106186808.046 |  |  | Within Groups | 47861214415.020 |  |
|  | Total | 112454586.122 |  |  | Total | 50785941629.379 |  |
| SHIELD only AQ4 GM noise intensity | Between Groups | 5399805.206 | 0.434 | SHIELD only AQ4 WM noise intensity | Between Groups | 291440814.049 | 0.478 |
|  | Within Groups | 72522042.690 |  |  | Within Groups | 4776295744.711 |  |
|  | Total | 77921847.896 |  |  | Total | 5067736558.760 |  |
| SHARD AQ4 GM noise intensity | Between Groups | 782696174.101 | 0.479 | SHARD AQ4 WM noise intensity | Between Groups | 1880435271.940 | 0.480 |
|  | Within Groups | 12904798641.289 |  |  | Within Groups | 31108852573.726 |  |
|  | Total | 13687494815.390 |  |  | Total | 32989287845.666 |  |
| SHARD+PB AQ4 GM noise intensity | Between Groups | 170801341.021 | 0.479 | SHARD+PB AQ4 WM noise intensity | Between Groups | 877902150.693 | 0.481 |
|  | Within Groups | 2822660952.292 |  |  | Within Groups | 14641723711.032 |  |
|  | Total | 2993462293.313 |  |  | Total | 15519625861.725 |  |
| SHIELD only tomato lectin GM normalized fraction volume | Between Groups | 1.107 | 0.100 | SHIELD only tomato lectin WM normalized fraction volume | Between Groups | 0.129 | 0.516 |
|  | Within Groups | 2.959 |  |  | Within Groups | 2.529 |  |
|  | Total | 4.066 |  |  | Total | 2.658 |  |
| SHARD tomato lectin GM normalized fraction volume | Between Groups | 0.508 | 0.379 | SHARD tomato lectin WM normalized fraction volume | Between Groups | 0.028 | 0.552 |
|  | Within Groups | 5.353 |  |  | Within Groups | 0.654 |  |
|  | Total | 5.862 |  |  | Total | 0.682 |  |
| SHIELD only GM GFAP normalized fraction volume | Between Groups | 0.001 | 0.405 | SHIELD only WM GFAP normalized fraction volume | Between Groups | 0.000 | 0.391 |
|  | Within Groups | 0.017 |  |  | Within Groups | 0.000 |  |
|  | Total | 0.019 |  |  | Total | 0.000 |  |
| SHARD GM GFAP normalized fraction volume | Between Groups | 0.489 | 0.608 | SHARD WM GFAP normalized fraction volume | Between Groups | 196.348 | 0.236 |
|  | Within Groups | 15.603 |  |  | Within Groups | 1096.185 |  |
|  | Total | 16.092 |  |  | Total | 1292.533 |  |
| SHIELD only AQ4 GM normalized fraction volume | Between Groups | 3.850 | 0.111 | SHIELD only AQ4 WM normalized fraction volume | Between Groups | 3.679 | 0.498 |
|  | Within Groups | 11.115 |  |  | Within Groups | 66.533 |  |
|  | Total | 14.965 |  |  | Total | 70.211 |  |
| SHARD AQ4 GM normalized fraction volume | Between Groups | 4.652 | 0.226 | SHARD AQ4 WM normalized fraction volume | Between Groups | 2.048 | 0.652 |
|  | Within Groups | 24.832 |  |  | Within Groups | 84.930 |  |
|  | Total | 29.484 |  |  | Total | 86.978 |  |
